# The adhesion GPCR GPR116/ADGRF5 has a dual function in pancreatic islets regulating somatostatin release and islet development

**DOI:** 10.1038/s42003-024-05783-9

**Published:** 2024-01-16

**Authors:** Juliane Röthe, Robert Kraft, Albert Ricken, Isabell Kaczmarek, Madlen Matz-Soja, Karsten Winter, André Nguyen Dietzsch, Julia Buchold, Marie-Gabrielle Ludwig, Ines Liebscher, Torsten Schöneberg, Doreen Thor

**Affiliations:** 1 Rudolf Schönheimer Institute of Biochemistry, Medical Faculty, Leipzig University, Leipzig, Germany; 2 Carl-Ludwig-Institute for Physiology, Medical Faculty, Leipzig University, Leipzig, Germany; 3 Institute of Anatomy, Medical Faculty, Leipzig University, Leipzig, Germany; 4 Medical Department II – Gastroenterology, Hepatology, Infectious Diseases, Pneumology, University Medical Center, Leipzig, Germany; 5 Division of Hepatology, Clinic and Polyclinic for Oncology, Gastroenterology, Hepatology, Infectious Diseases, and Pneumology, University Hospital, Leipzig, Germany; 6 Novartis Institutes for Biomedical Research, Basel, Switzerland

**Keywords:** Metabolism, Metabolic pathways

## Abstract

Glucose homeostasis is maintained by hormones secreted from different cell types of the pancreatic islets and controlled by manifold input including signals mediated through G protein-coupled receptors (GPCRs). RNA-seq analyses revealed expression of numerous GPCRs in mouse and human pancreatic islets, among them *Gpr116*/*Adgrf5*. GPR116 is an adhesion GPCR mainly found in lung and required for surfactant secretion. Here, we demonstrate that GPR116 is involved in the somatostatin release from pancreatic delta cells using a whole-body as well as a cell-specific knock-out mouse model. Interestingly, the whole-body GPR116 deficiency causes further changes such as decreased beta-cell mass, lower number of small islets, and reduced pancreatic insulin content. Glucose homeostasis in global GPR116-deficient mice is maintained by counter-acting mechanisms modulating insulin degradation. Our data highlight an important function of GPR116 in controlling glucose homeostasis.

## Abstract (web summary)

In vitro, ex vivo, and in vivo analysis shows that the Adhesion GPCR ADGRF5/GPR116 regulates islet functionality. Activation of the receptor leads to an increase in somatostatin secretion whereas loss of the receptor results in metabolic changes.

## Introduction

Pancreatic islets are composed of different cell types: endocrine cells, which secrete hormones required for maintaining glucose homeostasis, and non-endocrine cells such as endothelial cells and pericytes. Different endocrine cell types are distinguished within pancreatic islets. The insulin-secreting beta cells and the glucagon-secreting alpha cells represent about 80% and 15% of the islet cell population, respectively, whereas the 5% somatostatin-secreting delta cells constitute the third largest cell population in mouse and human pancreatic islets^1^. Because of the low amount of pancreatic islets and the short half-life time, it is assumed that somatostatin secreted from pancreatic delta cells only exhibits paracrine functions on other islet cell types^2,3^. Somatostatin receptors are expressed in beta and alpha cells suggesting that somatostatin prevents over-secretion of insulin and glucagon after nutrient stimulation^4^. In mouse islets, delta cells are mainly found in the islet’s periphery. However, delta cells have a neuron-like shape and, therefore, may reach a high number of other islet cell types for paracrine regulation^5^. So far, little is known about the regulation of somatostatin secretion besides nutrient-dependent release. It is well-established that G protein-coupled receptors (GPCRs) fine-tune hormone secretion in pancreatic alpha and beta cells^6^. Similar function has also been described for somatostatin release, e.g., by muscarinic acetylcholine M1 receptor activation^7^. Recently, it was demonstrated that the inhibitory effect of ghrelin on insulin secretion is mediated by somatostatin secreted from pancreatic delta cells^2,3^. Furthermore, a negative feedback effect on insulin secretion mediated by urocortin-3 and corticotropin-releasing hormone receptor 2 expressed on delta cells has been shown^8^. These recent results suggest that pancreatic delta cells are important for the paracrine regulation network in pancreatic islets.

Besides the endocrine cells, also endothelial cells have assigned functions in the regulation of islet functionality. Dysfunction of endothelial cells results in reduced insulin secretion and beta cell loss^9^. Several studies focused on the importance of endothelial cells on beta cell development. Thereby, it was shown that endothelial cells secrete growth factors driving beta cell proliferation^10^ and endocrine pancreatic differentiation^11^. Recent work demonstrated that islet GPCRs are also involved in those developmental processes^12–14^. However, the complete processes of islet development as well as differentiation towards the different endocrine cell types are not well understood.

Several studies have shown that the repertoire of GPCRs expressed in pancreatic islets is much broader than previously assumed^2,3,15,16^. Numerous adhesion GPCRs (aGPCRs) including latrophilins (LPHN1/ADGRL1, LPHN2/ADGRL2, and LPHN3/ADGRL3), GPR56/ADGRG1, and GPR116/ADGRF5 are among well-expressed GPCRs. Adhesion GPCRs form a separate class of GPCRs, which is widely uncharacterized regarding ligands and physiological functions mainly because of their extraordinary size^17^. A milestone in investigating their physiological function was the discovery that these receptors can be activated by a tethered agonist^18,19^.

GPR116 was initially described as an unusual GPCR with a large N terminus composed of immunoglobulin domains^20^. It was also demonstrated that GPR116 carries an internal agonist and peptides derived from this sequence can be used for receptor activation in vitro and ex vivo^21,22^. GPR116 is highly expressed in lung endothelial cells regulating surfactant secretion and its deficiency results in lipid and protein accumulation as well as macrophage activation and inflammation in the alveols^23–26^. Besides lung tissue, GPR116 is abundantly expressed in endothelial cells involved in the vasculature development of the nervous system and in cardiovascular functions^27,28^. Moreover, several studies have described involvement of GPR116 in carcinogenesis and cancer metastasis^29–32^. Adipocyte-specific knock-out (ko) of GPR116 suggested a role in metabolic processes^33,34^.

In the present study, we show that GPR116 is expressed in pancreatic delta cells modulating somatostatin secretion upon activation. Furthermore, GPR116 deficiency reduces the size and number of beta cells and, subsequently, the insulin content of the endocrine pancreas. Our data demonstrate an important role of GPR116 in glucose homeostasis necessary for assembling fully functional pancreatic islets.

## Results

### GPR116 is expressed in pancreatic islets

**Fig. 1:**
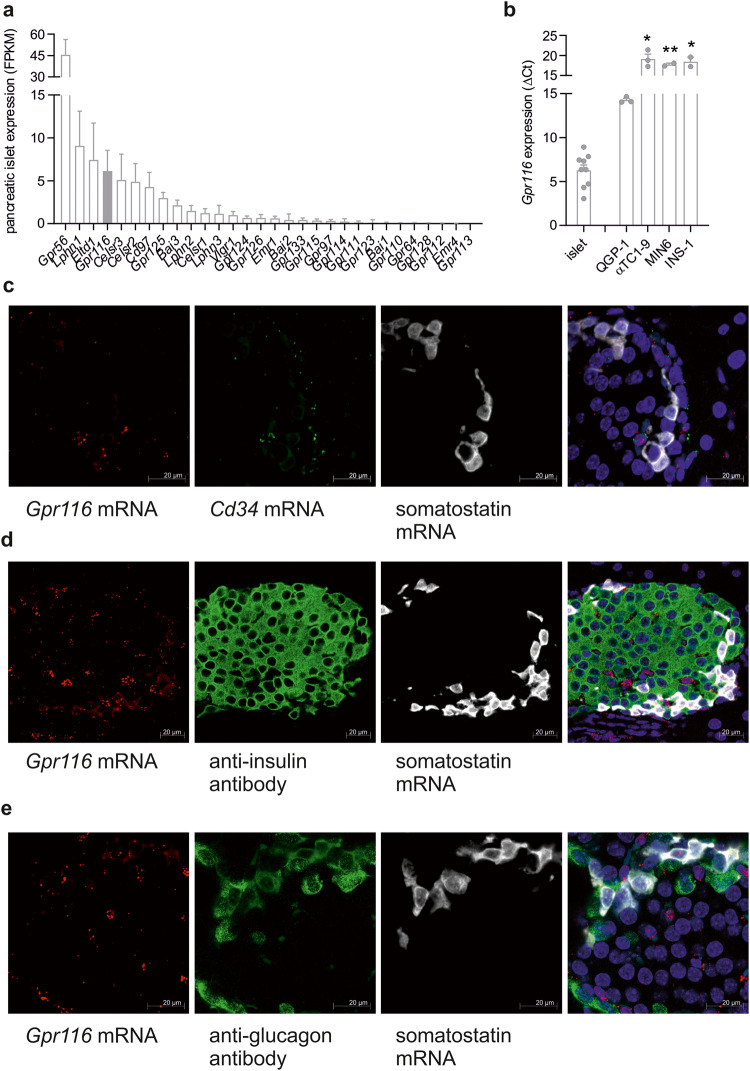
Expression of GPR116 and structurally related aGPCRs in pancreatic islet cells. **a** Pancreatic islet RNAseq analysis^16^ was re-analyzed to determine the expression of aGPCRs. Here, we observe high expression of aGPCRs with an already described function in islets such as *Gpr56* or *Lphn1*. Among the highly expressed receptors we also detected *Gpr116*. Given is the mean ± SD of 10 animals. **b** qPCR analysis was performed to confirm high expression of *Gpr116* in pancreatic islets. Furthermore, we also detected expression in specific cell lines mimicking delta (QGP-1), alpha (αTC1-9) and beta (MIN6, INS-1) cells. Given is the relative expression of GPR116 normalized to β-actin as reference gene as mean ± SEM of 2 (INS-1, MIN6), 3 (QGP-1, αTC1-9), or 9 (islets) independent experiments carried out in triplicates. Statistical differences in expression were determined in the cell lines αTC1-9, MIN6, and INS-1 in comparison to QGP-1 cells using Student’s *t* test (*P ≤ 0.05; **P ≤ 0.01). **c**–**e** To detect cell-type specific expression of *Gpr116* mRNA in situ hybridization studies combined with immunofluorescence was performed on sections of mouse pancreas. Probes for *Gpr116* (red) and somatostatin (white) as well as *Gpr116* (red) and *Cd34* (green) showed a partially overlapping expression (**c**), while *Gpr116* mRNA was hardly detected in insulin- (**d**), or glucagon-positive (**e**) cells shown in green.

Re-analysis of own RNA-sequencing (RNA-seq) data^16^ of mouse pancreatic islets revealed similarly high expression of several aGPCRs as reported for other receptors modulating insulin and glucagon secretion (Fig. 1a). Among these, pancreatic functions of the aGPCRs *Gpr56*, *Lphn1*, *Bai3*, and *Lphn3* have already been shown^35–38^. Another aGPCR, *Gpr116*, has been implicated in metabolic processes^33,34^, but not specifically in pancreatic islet function. Quantitative PCR (qPCR) analysis confirmed the abundant expression of *Gpr116* in mouse pancreatic islets (Fig. 1b). To test for cell type-specific expression of *Gpr116*, we performed analysis of different cell lines representing alpha (αTC1-9), beta (MIN6, INS-1), and delta (QGP-1) cells derived from pancreatic islets (Fig. 1b). Interestingly, we found highest expression in the delta cell line QGP-1, whereas expression in αTC1-9, MIN6, and INS-1 cells was significantly lower. This is in line with two published RNA-seq data sets of sorted mouse pancreatic islet cells^2,3^, which we re-analyzed to compare the expression of *Gpr116* in alpha, beta, and delta cells (Supplementary Experimental Procedures). Data from both sources revealed a significantly enriched expression in delta cells (Supplementary Fig. 1a).

To confirm the expression of *Gpr116* in delta cells we used in situ hybridization combined with antibody staining in pancreas sections (Fig. 1c–e). Here, *Gpr116* expression was clearly visible in somatostatin-expressing cells, but only in a small amount of insulin- and glucagon-positive cells (Fig. 1d, e). Additionally, *Gpr116* expression was observed in an additional subset of islet cells co-expressing *Cd34* (Fig. 1c) indicating its presence in endothelium cells.

### GPR116 signals via Gα_q/11_ proteins inducing Ca^2+^ transients in pancreatic islets

GPR116 belongs to the subfamily VI of aGPCRs, which includes the structurally related GPR110, GPR111, and GPR115^39^. *Gpr110*, *Gpr111*, and *Gpr115* exhibited only minor expression in pancreatic islets (Supplementary Fig. 1b). Similar to other aGPCRs^18^, a peptide (p116) derived from an intramolecular amino acid sequence, the so-called *Stachel* sequence, has been demonstrated to activate GPR116^21,22^. Prior to the activation of endogenously expressed GPR116, the agonistic property of p116 was verified in different control experiments (see Supplementary Results and Supplementary Fig. 1c–g). To control for unspecific peptide effects, we used a scrambled peptide (p116sc) as control in all experiments.

**Fig. 2:**
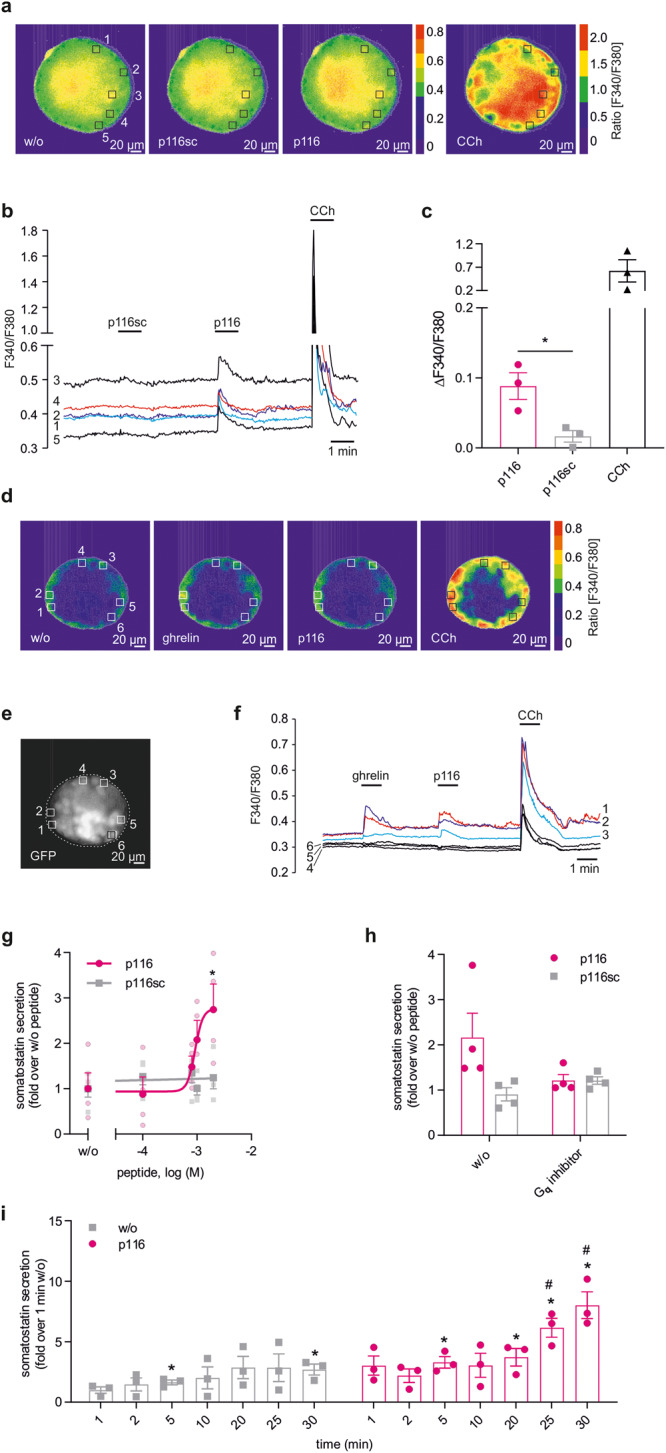
GPR116 signaling in mouse pancreatic islets. **a** Ca^2+^ imaging experiments were performed on single pancreatic islets from C57BL6/N mice loaded with fura-2 AM. Images show the fluorescence ratio (F340/F380) of one representative islet before and after application of 1 mM p116sc, 1 mM p116, and 100 µM carbachol (CCh). **b** Time course of Ca^2+^ responses in five representative regions (1–5) within the islet is shown and corresponds to **c**. **c** Changes in intracellular Ca^2+^ concentration induced by p116 (1 mM), p116sc (1 mM), and CCh (100 µM) in pancreatic islets. Relative changes in the Ca^2+^ concentration are presented as mean delta ratio (F340/F380) ± SEM from three islets containing five to seven regions of interest (defined as squares of 13 µm side length). Statistical significance was tested using a two-tailed unpaired *t* test (*P ≤ 0.05). **d** Fura-2-based Ca^2+^ imaging experiments were performed on single pancreatic islets from MIP-GFP mice, expressing Enhanced Green Fluorescent Protein (EGFP) under the control of the mouse insulin 1 promoter. Images show the fluorescence ratio (F340/F380) of one representative islet before and after application of ghrelin (300 nM), p116 (1 mM), and carbachol (CCh, 100 µM). **e** Six representative regions (1–6) within the islet were chosen showing EGFP fluorescence of low (regions 1–3) or moderate (regions 4–6) intensity. These regions correspond to those shown in **d**. **f** Time course of Ca^2+^ responses in regions 1–3 (colored) and 4–6 (black) within the islet corresponds to **d**. **g** Somatostatin secretion from pancreatic islets of wt C57BL6/N mice was measured after incubation (30 min) with different concentrations of GPR116 agonist p116 and inactive peptide p116sc (control) under high-glucose (16.7 mM) conditions. Given is the mean ± SEM as fold over non-stimulated islets (1.9 ± 0.5 ng/ml) of four independent experiments performed in duplicates. Statistical significance was tested using one-way ANOVA with Dunnet’s multiple comparison test. *P ≤ 0.05 compared with non-stimulated islets. **h** After pre-incubation with the G_q_ inhibitor FR900359 pancreatic islets were incubated with 2 mM p116 or 2 mM p116sc and somatostatin secretion was measured under high-glucose conditions after 30 min. Data are shown as fold over somatostatin secretion of non-stimulated islets (w/o G_q_ inhibitor: 2.0 ± 0.6 ng/ml; G_q_ inhibitor: 1.4 ± 0.2 ng/ml), given as mean ± SEM of four independent experiments carried out in duplicates. **i** Somatostatin secretion from wt pancreatic islets (C57BL6/N) was measured under high-glucose conditions with and without 2 mM p116 at different time points. Shown is the mean ± SEM as fold over 1 min glucose-stimulated islets (basal somatostatin secretion: 0.6 ± 0.2 ng/ml) of three independent experiments performed in duplicates. *P* values were determined using one-way ANOVA. *compared to non-stimulated islets 1 min after incubation; # compared to p116 stimulated islets 1 min after incubation (*P ≤ 0.05).

Next, the signaling properties of endogenous GPR116 were investigated in isolated primary islets. Islets were exposed to p116 (specific agonist), p116sc (peptide control), and carbachol (CCh, agonist of muscarinic acetylcholine receptors and established positive control), and Ca^2+^ responses were imaged. As expected, CCh application induced a strong Ca^2+^ elevation in most parts of the islets (Fig. 2a). Ca^2+^ transients were also elicited by p116, but appeared more scattered within the islets (Fig. 2a). The control peptide p116sc did not induce significant Ca^2+^ responses (Fig. 2a, b). Quantification of Ca^2+^ signals induced by p116 or p116sc within responsive regions revealed a specific activation of GPR116 in distinct pancreatic islet cells (Fig. 2c). Stimulation with ghrelin which activates the G_q/11_ protein-coupled growth hormone secretagogue receptor/ghrelin (GHS-R) receptor^40^, and subsequent application of p116 elicited Ca^2+^ signals with partially overlapping localization (Fig. 2d). Ghrelin-induced Ca^2+^ signals can be used to identify pancreatic delta cells in primary islets^41^ and responses upon both, ghrelin and p116, were absent in insulin-expressing beta cells (Fig. 2e, f). Therefore, the lack of GPR116-mediated Ca^2+^ signals in beta cells is in line with the expression pattern of *Gpr116*.

Taken together, our results demonstrate that GPR116 is also functionally expressed in a subset of pancreatic islet cells.

### GPR116 regulates somatostatin secretion from pancreatic islets

To test functionality of *Gpr116* expression in delta cells, we determined somatostatin secretion upon GPR116 activation under high-glucose conditions. As shown in Fig. 2g, secretion of somatostatin is significantly increased (2.7-fold at 2 mM p116 yielding an EC_50_ of 950 µM ± 124 µM) after incubation of islets with p116, whereas p116sc did not induce secretion. To test whether the p116-induced somatostatin release is mediated via activation of the Gα_q/11_ protein/phospholipase C signaling pathway, pancreatic islets were incubated with the G_q_ inhibitor FR900359^42^ which completely abolished somatostatin secretion upon p116 application (Fig. 2h). Time-dependent measurement of somatostatin release revealed an immediate (within 1 min) and a prolonged increase (within 30 min) of somatostatin secretion after stimulation with p116 under high-glucose conditions (Fig. 2i). Whereas the GPR116-independent release showed a steady increase within 30 min, p116 caused a significant increase already after 5 min of incubation (Fig. 2i). These data demonstrate that activation of GPR116 augments glucose-induced somatostatin secretion over a time period of 30 min.

Taken together, our results identify GPR116 as a novel receptor modulating somatostatin secretion from pancreatic delta cells.

### Islet from GPR116-deficient mice display a lack of p116-induced somatostatin release and a reduced insulin content

**Fig. 3:**
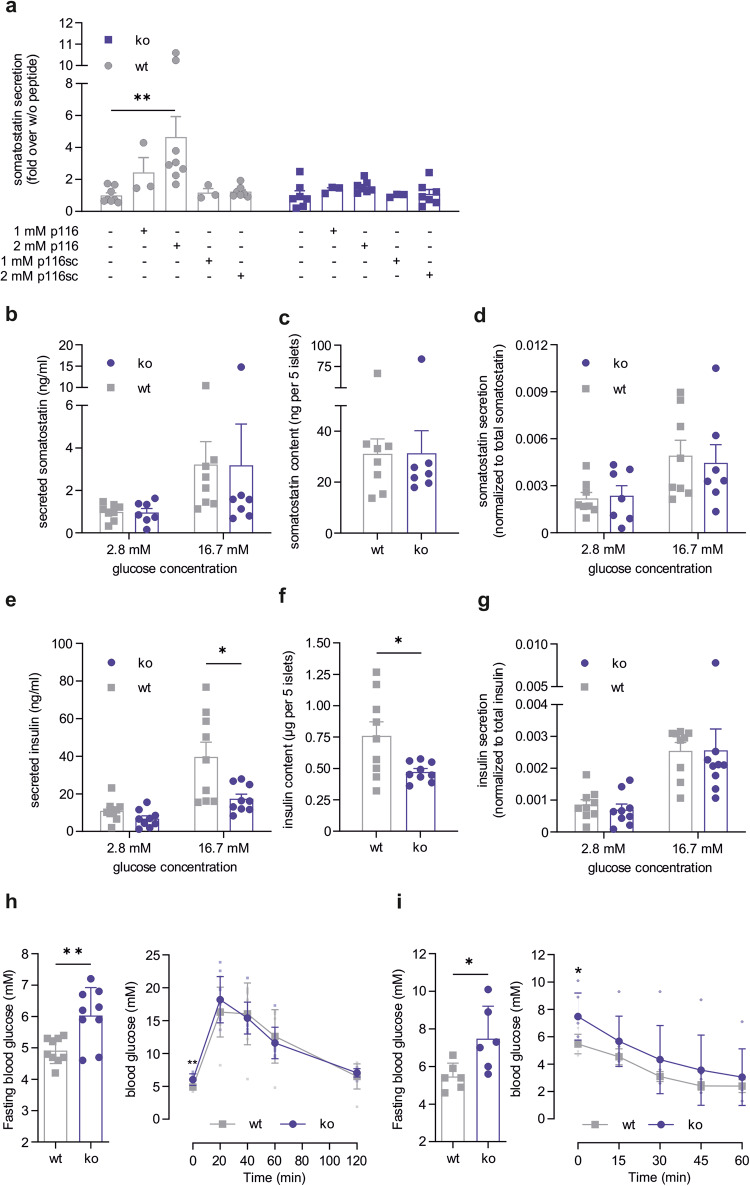
Constitutive GPR116 ko mice show reduced insulin secretion and decreased insulin content in pancreatic islets. **a** Somatostatin secretion from pancreatic islets of wt and ko mice was measured after incubation with different concentrations of p116 (agonist) and p116sc (control). Given is the mean ± SEM fold over non-stimulated islets (wt: 2.7 ± 1.0 ng/ml; ko: 2.8 ± 1.7 ng/ml) of *n* = 3 (1 mM peptide) or *n* = 7 or 8 (non-stimulated and 2 mM peptide of ko and wt, respectively) performed in duplicates. *P* values were determined using one-way ANOVA in comparison to the respective non-stimulated incubation (**P ≤ 0.01). **b**–**d** Analysis of glucose-induced somatostatin secretion. Secreted somatostatin (**b**) as well as somatostatin content (**c**) from wt and ko pancreatic islets were quantified at low (2.8 mM) and high (16.7 mM) glucose conditions. **d** For each sample, ratios for glucose-induced somatostatin secretion were calculated as a percentage of total somatostatin content. Data are presented as mean ± SEM performed in duplicates of *n* = 8 wt and *n* = 7 ko and 5 islets per sample. **e**–**g** Analysis of glucose-induced insulin secretion. Secreted insulin (**e**) as well as insulin content (**f**) from wt and ko pancreatic islets were quantified at low (2.8 mM) and high (16.7 mM) glucose conditions. **g** For each sample, ratios for glucose-induced insulin secretion were calculated as a percentage of total somatostatin content. Data are presented as mean ± SEM performed in duplicates of *n* = 9 and 5 islets per sample. *P* values were determined using an unpaired multiple *t* test (Holm-Šídák method) (*P ≤ 0.05). **h** Blood glucose was measured in overnight fasted ko and wt mice. Given is the mean glucose level ± SD performed in triplicates for *n* = 9 mice. Statistical significance was tested using a two-tailed unpaired *t* test (**P ≤ 0.01). Glucose tolerance test was performed by measuring blood glucose levels at different time points after peritoneal glucose injection. Data represent mean ± SD of 9 mice per genotype. **i** Blood glucose was measured after 6-hour fasting in ko and wt mice. Given is the mean glucose level ± SD for 6 animals per genotype. Statistical significance was tested using a two-tailed unpaired *t* test (*P ≤ 0.05). Insulin tolerance test was performed in by measuring blood glucose levels at different time points after peritoneal insulin injection. Data represent mean ± SD of 6 mice per genotype.

To prove that the peptide-induced somatostatin secretion is mediated by GPR116, we used a previously established germline ko mouse strain with a complete deletion of exon 17 (exon annotation based on the mouse GRCm38/mm10 assembly) resulting in a non-functional receptor^23^ (Supplementary Results and Supplementary Figs. 2a–d). Thus, we measured GPR116-induced somatostatin secretion from wt and ko pancreatic islets. As expected, somatostatin secretion from wt islets was increased after p116 stimulation in a concentration-dependent manner whereas p116sc did not have a significant effect (Fig. 3a). In GPR116 ko islets, p116 was ineffective to liberate somatostatin even at high peptide concentration (2 mM) indicating that peptide-induced somatostatin secretion depends on the expression of a functional GPR116 (Fig. 3a).

Next, we investigated the amounts of somatostatin and insulin secreted from isolated pancreatic islets of ko and wild-type (wt) mice after incubation with low- and high-glucose solution. We observed no changes in glucose-induced somatostatin secretion between wt and ko pancreatic islets (Fig. 3b). Interestingly, the amount of insulin released from ko pancreatic islets was slightly decreased under low-glucose conditions and significantly reduced under high-glucose conditions compared to wt islets (Fig. 3e). Moreover, while the content of somatostatin was unchanged in ko compared to wt islets (Fig. 3c), the total amount of insulin was significantly reduced in ko pancreatic islets (60% of wt islets) (Fig. 3f). However, relative glucose-induced somatostatin and insulin secretion, normalized to the total amounts of the respective hormones, were not changed in ko pancreatic islets compared to wt islets (Fig. 3d, g).

In general, the ko mice did not display an obvious metabolic phenotype. Neither male nor female mice showed a difference in body weight at an age of 14 to 16 weeks (Supplementary Fig. 2e). To analyze the physiological impact of the reduced insulin secretion onto glucose homeostasis in constitutive GPR116 ko animals, glucose tolerance tests were performed in overnight-fasted wt and ko mice. We found significantly increased blood glucose levels after overnight fasting in GPR116 ko mice compared to wt animals (Fig. 3h). However, no obvious differences between wt and ko mice were detected in glucose tolerance tests (Fig. 3h). Insulin tolerance tests, performed after 6 h fasting, also displayed no significant differences between wt and ko mice despite an initial difference in fasting blood glucose levels (Fig. 3i).

Our data demonstrates that the p116-induced somatostatin secretion is specific for GPR116 and that the receptor is required for adequate insulin production and, as a result, for sufficient glucose-induced insulin secretion. However, the reduced amount of insulin does not result in an impaired glucose tolerance.

### Delta cell-specific GPR116 activation mediates somatostatin release

**Fig. 4:**
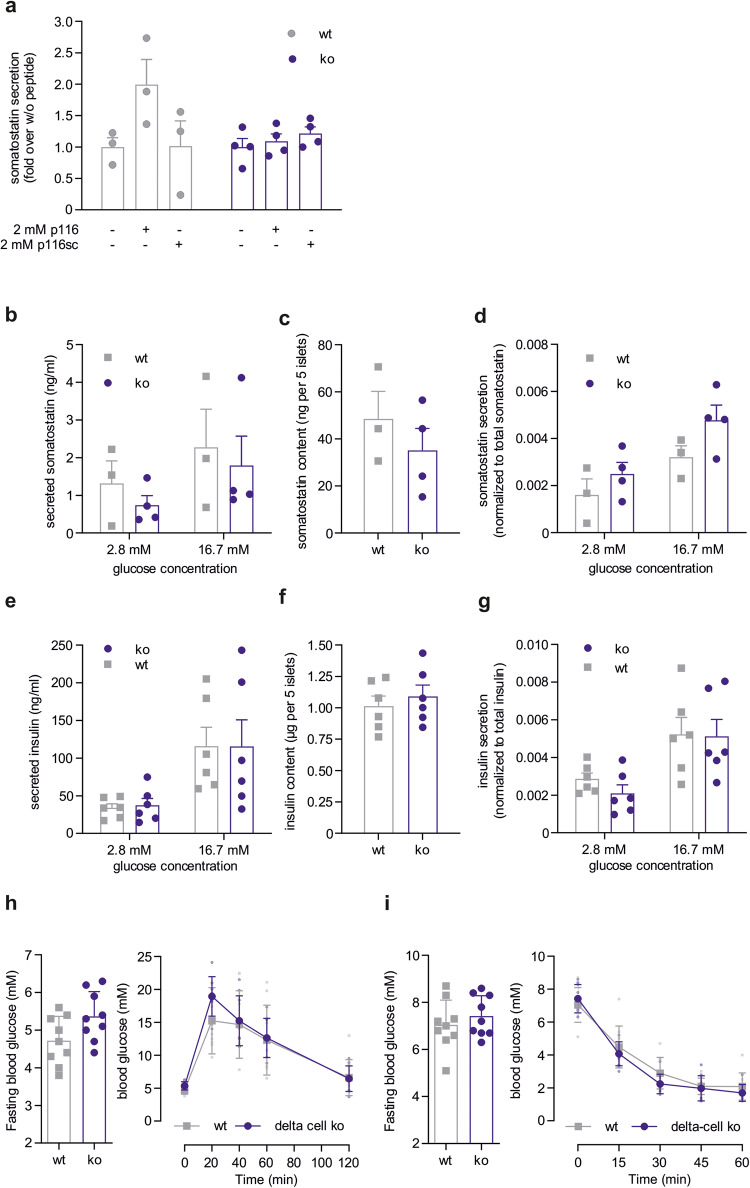
Characterization of delta cell-specific *Gpr116* ko mice. **a** Somatostatin secretion from pancreatic islets of wt and delta cell-specific ko mice was measured after incubation with p116 (agonist) or p116sc (control). Given is the mean ± SEM fold over non-stimulated islets of *n* = 4 (wt) or 4 (ko) performed in duplicates. Statistical significance was tested using a two-tailed unpaired *t* test (*P ≤ 0.05). **b**–**d** Analysis of glucose-induced somatostatin secretion. Secreted somatostatin (**b**) as well as somatostatin content (**c**) from wt and delta cell-specific ko pancreatic islets were quantified at low (2.8 mM) and high (16.7 mM) glucose conditions. **d** For each sample, ratios for glucose-induced somatostatin secretion were calculated as a percentage of total somatostatin content. Data are presented as mean ± SEM performed in duplicates of *n* = 3 (wt) or 4 (ko). **e**–**g** Analysis of glucose-induced insulin secretion. Secreted insulin (**e**) as well as insulin content (**f**) from wt and delta cell-specific ko pancreatic islets were quantified at low (2.8 mM) and high (16.7 mM) glucose conditions. **g** For each sample, ratios for glucose-induced insulin secretion were calculated as a percentage of total somatostatin content. Data are presented as mean ± SEM performed in duplicates of *n* = 6 and 5 islets per sample. **h** Blood glucose was measured in overnight fasted delta cell-specific ko and wt mice. Given is the mean glucose level ± SD performed in triplicates for *n* = 9 mice. Glucose tolerance test was performed by measuring blood glucose levels at different time points after peritoneal glucose injection. Data represent mean ± SD of 9 mice per genotype. **i** Blood glucose was measured after 6-hour fasting in delta cell-specific ko and wt mice. Given is the mean glucose level ± SD for 9 animals per genotype. Insulin tolerance test was performed in by measuring blood glucose levels at different time points after peritoneal insulin injection. Data represent mean ± SD of 9 mice per genotype.

To specify the role of GPR116 in pancreatic delta cells, *Gpr116*^f/f^ ^23^ mice were crossed to Sst-IRES-Cre transgenic mice (Jackson Laboratories, #018973). Islets isolated from delta cell-specific ko animals show transcripts of *Gpr116* with deleted exon 17 and a reduced amount of full-length receptor (Supplementary Fig. 2f, g) indicating the feasibility of this approach. Furthermore, using the specific GPR116 peptide agonist p116, we could trigger somatostatin release from wt pancreatic islets, however, delta cell-specific ko of GPR116 completely abolished this signal (Fig. 4a).

When analyzing glucose-induced somatostatin and insulin secretion, we found that deletion of functional GPR116 in pancreatic delta cells neither changed the amounts of secreted hormones nor the content (Fig. 4b, c for somatostatin, Fig. 4e, f for insulin). Furthermore, glucose responsiveness evaluated by the normalized values was comparable for wt and ko animals for both hormones (Fig. 4d, g).

This is also reflected in vivo with no major differences when performing glucose and insulin tolerance tests (Fig. 4h, i).

Using a delta cell-specific ko mouse model we demonstrate that activation of GPR116 expressed in pancreatic delta cells increases somatostatin release. However, the observed reduced insulin content in pancreatic islets as well as the increased fasting blood glucose levels of constitutive GPR116-deficient mice is not caused by the absence of GPR116 expressed in delta cells.

### GPR116 deficiency reduces insulin secretion in vivo and alters insulin metabolization

**Fig. 5:**
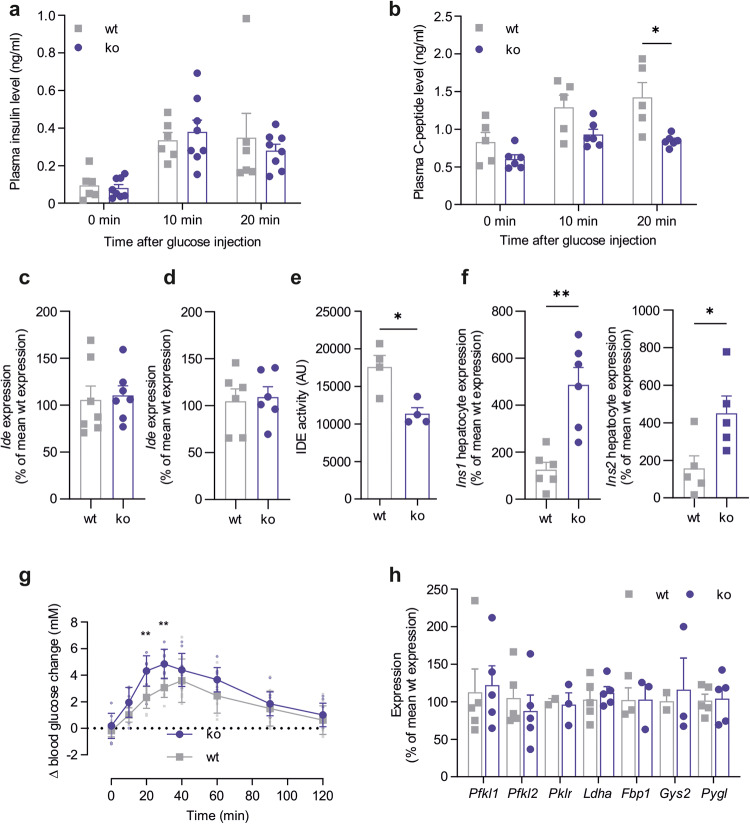
Analysis of insulin metabolism in constitutive GPR116 ko mice. **a** Plasma insulin concentration in samples of wt (*n* = 6) and ko (*n* = 8) mice was determined at different time points after peritoneal glucose injection with the mouse insulin ELISA. Given are plasma insulin concentrations as mean ± SEM performed in duplicates. **b** C-peptide concentration in samples of wt (*n* = 5) and ko (*n* = 6) mice was determined at different time points after peritoneal glucose injection with the mouse C-peptide ELISA. Given are plasma concentrations as mean ± SEM performed in duplicates. *P* values were determined using an unpaired multiple *t* test (Holm-Šídák method) (*P ≤ 0.05). **c**, **d** Expression of insulin degrading enzyme *Ide* is not changed by *Gpr116* ko neither in liver (**c**) nor in hepatocytes (**d**). Given is the percentage of wt expression in reference to *Actb* expression as mean ± SEM of *n* = 7 (wt) or *n* = 6 (ko) each performed in triplicates. **e** IDE activity was analyzed in hepatocytes isolated from wt and constitutive GPR116 ko mice using a SensoLyte IDE activity assay. Given is the mean ± SEM of 4 independent experiments each performed in duplicates. Statistical significance was tested using a two-tailed unpaired *t* test (*P ≤ 0.05). **f** Expression levels of *Ins1* and *Ins2* were determined in isolated hepatocytes from wt and ko mice. Data are presented as percentage of wt expression in reference to *Actb* expression and given is the mean ± SEM of *n* = 6 (*Ins1*) and *n* = 5 (*Ins2*) each performed in triplicates. Statistical significance was tested using a two-tailed unpaired *t* test (*P ≤ 0.05; **P ≤ 0.01). **g** Pyruvate tolerance test was performed in BALB/c wt and ko mice by measuring blood glucose levels at different time points after peritoneal pyruvate injection. Data represent mean ± SD of 9 mice per genotype. *P* values were determined using an unpaired multiple *t* test (Holm-Šídák method) (**P ≤ 0.01). **h** Expression analysis of key enzymes of glycolysis, gluconeogenesis or glycogen metabolism in hepatocytes reveals no differences between wt and ko animals. Data were obtained using qPCR, are normalized to *Actb* expression, and are shown as percentage of wt expression. Given is the mean ± SEM of 2–5 wt and ko mice.

Constitutive GPR116 ko leads to increased fasting blood glucose levels already under chow diet (see above). To investigate the mechanisms resulting in this phenotype, we further evaluated metabolic characteristics of these ko animals. To analyze whether the reduced islet insulin secretion is also observed in vivo, we measured plasma insulin content in overnight fasted ko and wt mice at 0, 10, and 20 min after intraperitoneal injection of glucose (Fig. 5a) and no significant differences were observed. However, when analyzing C-peptide plasma concentration, we found significantly reduced amounts after 20 min of glucose administration (Fig. 5b) indicating that the reduced islet insulin secretion also leads to a reduced amount of released insulin in vivo. As this did not translate into different plasma insulin levels, we speculated that insulin metabolization might be affected by GPR116 ko. The insulin-degrading enzyme (IDE) is expressed in hepatocytes and functions as the key enzyme in insulin degradation. Thus, we analyzed *Ide* expression in liver tissue (Fig. 5c) and isolated hepatocytes (Fig. 5d), however, no differences were observed. Next, we determined activity of IDE in hepatocytes isolated from ko and wt mice and detected a reduced activity in GPR116 ko animals (Fig. 5e). Furthermore, insulin expression was significantly higher in hepatocytes isolated from ko animals compared to wt siblings (Fig. 5f). Together, reduced degradation and increased production of insulin in hepatocytes could be responsible for adjusting plasma insulin levels as a result of dysregulated glucose levels. Indeed, we found an increased insulin expression in isolated wt hepatocytes when stimulated with high glucose (Supplementary Fig. 3a).

Since differences in fasting blood glucose levels hint towards changes in liver gluconeogenesis, we performed pyruvate tolerance tests in overnight-fasted animals. Here, ko mice showed a significant increase in blood glucose levels 20 and 30 mins after pyruvate injection indicating a higher gluconeogenesis rate compared to wt animals (Fig. 5g). Therefore, we analyzed expression of key enzymes of glycolysis and gluconeogenesis in liver samples of wt and ko mice but found no differences (Fig. 5h).

### GPR116 ko alters pancreatic islets

**Fig. 6:**
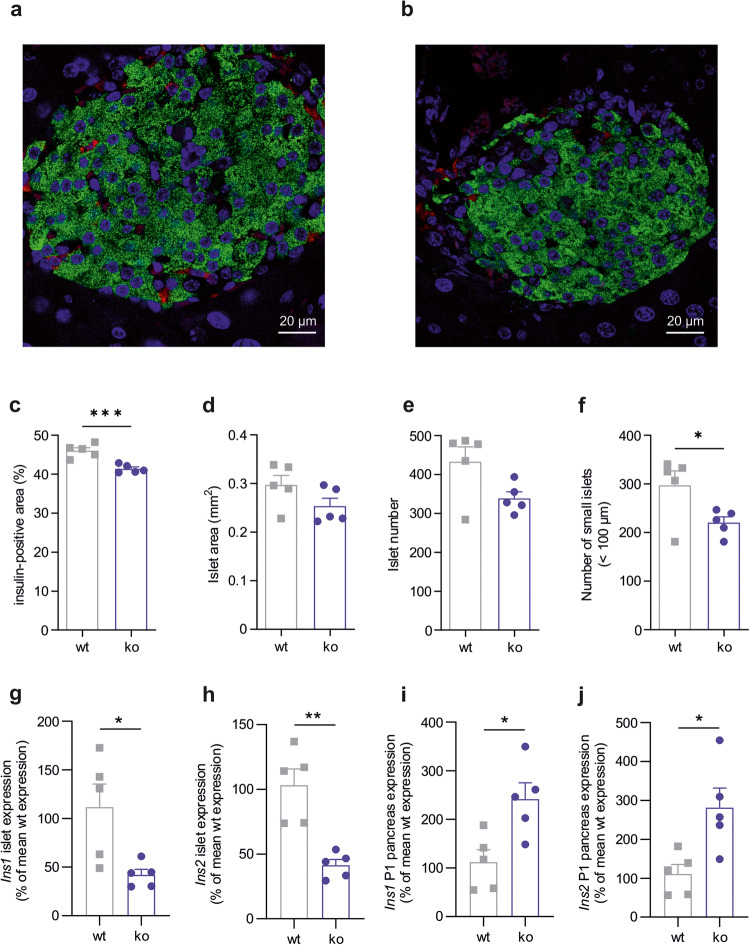
GPR116 deficiency affects islet number, insulin-positive area, and insulin expression. **a**, **b** Immunofluorescence staining of mouse pancreatic islets of wt (**a**) and ko (**b**) mice. Shown are representative images of wt and ko islets with insulin-positive cells (green), somatostatin-positive cells (red), and DAPI-stained nuclei (blue). **c** Beta-cell amount was calculated as percentage of insulin-positive cell area relative to the total pancreatic islet area in pancreatic sections of wt and GPR116 ko mice. Shown is the mean ± SEM of all sections of five wt and ko mice. **d** The islet area was quantified in all analyzed sections of five wt and ko BALB/c animals. Data is given as mean ± SEM. **e** The total number of islets was counted in all pancreatic sections and is shown as mean ± SD of *n* = 5 per genotype. **f** The diameter of stained islets was calculated to classify islet size. Number of islets (<100 µm) were counted and are displayed as mean ± SD of all sections of *n* = 5 per genotype. **g**, **h** Insulin expression (*Ins1* and *Ins2*) was determined in adult islets from 5 wt and ko animals. Data is given as percentage of wt expression (mean ± SEM) normalized to expression of *Actb* as reference gene. **i**, **j** Insulin expression (*Ins1* and *Ins2*) was determined in pancreas of P1 animals (*n* = 5 per genotype). Data is given as percentage of wt expression (mean ± SEM) normalized to expression of *Actb* as reference gene. Statistical significance was tested using a two-tailed unpaired *t* test (*P ≤ 0.05; **P ≤ 0.01; ***P ≤ 0.001).

The previous results show that deletion of GPR116 specifically in pancreatic delta cells is not the cause for the observed change in insulin content of constitutive GPR116 ko animals. To find the cause of this phenotype, we quantified the amounts of insulin-positive beta cells and somatostatin-positive delta cells. Thereto, we performed immunofluorescence staining of sections of mouse pancreas with specific antibodies against insulin and somatostatin (Fig. 6a, b). The analysis revealed a decreased number of insulin-positive beta cells in GPR116 ko mice while the amount of somatostatin-positive delta and glucagon-positive alpha cells did not significantly differ between wt and ko mice (Fig. 6c and Supplementary Fig. 3b, c).

Furthermore, trends towards smaller total islet area in pancreatic sections and to a decreased total number of pancreatic islets in ko mice were observed (Figs. 6d and 6e). In-depth analysis showed a significantly reduced number in small islets with a diameter of < 100 µm (Fig. 6f and Supplementary Fig. 3d).

Quantitative PCR analysis of adult mice revealed that expression of the insulin genes *Ins1* and *Ins2* was significantly lower in ko islets compared to wt islets (Fig. 6g, h). Also, somatostatin expression is significantly reduced in ko islets compared to wt islets, whereas glucagon expression remained unchanged (Supplementary Fig. 3e, f). Interestingly, we observed the opposing expression pattern in pancreatic tissue from neonatal P1 mice. Here, ko animals showed significantly higher insulin gene expression (Fig. 6i, j). This data suggests that GPR116 is already involved in the development of pancreatic islets leading to a reduced amount of beta cells as well as pancreatic islets.

## Discussion

Mammalian pancreatic islets consist of five different endocrine cell types - alpha, beta, delta, epsilon, and PP cells^43^ - as well as endothelial cells^44^. While the most abundant insulin- and glucagon-secreting beta and alpha cells have been intensively studied, less attention has been paid to the other cell types. However, paracrine crosstalk between pancreatic cells may influence islet cell composition and hormone secretion. For example, it is well established that somatostatin released by pancreatic delta cells negatively regulates both, insulin and glucagon secretion^45,46^. Currently, the importance of somatostatin secreted by delta cells is discussed with respect to insulin-induced hypoglycemia in diabetic models^47^. Furthermore, interactions of pancreatic beta cells and endothelial cells have been proposed^48^.

Here, we describe expression of GPR116, a member of the adhesion GPCR class, in pancreatic islets. Besides its well-studied function in lung surfactant regulation, GPR116 is also involved in endothelial function and metabolic processes^23–25,27,33,34^. However, due to its high expression in pancreatic islets^2,3,15,16^, we assumed a role of GPR116 in metabolic processes beyond modulating insulin sensitivity in adipocytes^33,34^. In contrast to rhodopsin-like GPCRs the role of aGPCRs in pancreatic islets is not well-established, however, some aGPCRs have been shown to regulate islet function and/or hormone secretion. GPR56 has been implicated in beta cell viability and insulin secretion in pancreatic islets via collagen III interaction^35,36^. LPHN3 and BAI3 activation have been linked to reduction of insulin secretion^37,38^, whereas the activation of LPHN1 increases the release of insulin^37^. Because most aGPCRs are still orphan receptors, elucidating their physiological roles is severely limited. Previous work on GPR116 identifying synthetic peptides derived from the *Stachel* sequence of the receptor suitable for activation^21,22^ and a knock-out mouse line enabled us to study the function of GPR116 in pancreatic islets.

The here presented data clearly demonstrate that abundant *Gpr116* expression is found in somatostatin-positive islet cells, while there is only marginal overlap with insulin or glucagon (Fig. 1) suggesting a role in pancreatic delta cells. This is supported by RNA-seq data of sorted pancreatic cells^2,3^ indicating a high expression of GPR116 in pancreatic delta cells but not alpha or beta cells. According to a delta cell-dependent function we found that activation of GPR116 using a *Stachel*-derived peptide results in a robust somatostatin release which could be blocked using the Gα_q/11_ inhibitor FR900359^42^ (Fig. 2g, h) indicating that the effect of somatostatin release is G_q/11_-protein dependent. These data confirm the reported GPR116-mediated G-protein signaling in vitro and ex vivo^21,22,32^. Because of the observation that GPR116 is expressed by another islet cell type, we used a constitutive and delta cell-specific *Gpr116* ko mouse model^23^ to demonstrate that the modulation of somatostatin secretion is mediated by delta-cell GPR116 (Figs. 3a and 4a). Therefore, we conclude that activation of GPR116 induces somatostatin release via a G_q/11_ protein/Ca^2+^-mediated mechanism. Thus, GPR116-dependent regulation of somatostatin secretion shows similarity to the signaling of the ghrelin receptor expressed in delta cells^2,3^. Unfortunately, the endogenous signal for triggering somatostatin release via GPR116 still remains unknown. Recently, the hepatokine FNDC4 has been identified to activate GPR116 leading to an increase in intracellular cAMP^34^. However, the increase of somatostatin secretion is a result of the G_q_ protein pathway which is not activated via the FNDC4 / GPR116 axis. It is tempting to speculate that the immunoglobulin-like domains of the large N terminus of GPR116 mediates GPR116 activation by cell-cell contacts. Also, aGPCRs have been shown to be activated by cell-matrix molecules like collagen and laminin^49–51^ or by mechanical forces^51–53^. Even though there has no mechano-activation of GPCRs been shown on pancreatic islets, it has been reported that glucose stimulation might cause an increase in volume and, therefore, membrane tension in pancreatic cells^54,55^.

Interestingly, using the constitutive knock-out of GPR116 we observed several changes regarding the pancreatic islets which are not connected to the receptor’s function in delta cells. Such, insulin content which was significantly reduced in GPR116 ko islets leading to a reduced glucose-stimulated insulin secretion. However, relative insulin secretion (in relation to insulin content) was not different in wt and ko mice (Fig. 3), indicating no alteration of the secretion process itself. Furthermore, ko animals display increased fasting blood glucose already under chow diet. This phenotype was also not observed using the delta-cell specific ko mouse model. Despite the reduced amount of secreted insulin, we did not observe altered glucose or insulin tolerances when comparing wt and ko mice. This seems to be contrary to previous work, where an adipocyte-specific GPR116 ko resulted in an impaired glucose tolerance^33,34^. However, a germline GPR116 ko may mask this adipocyte-specific phenotype because of summative results in the regulation of the glucose homeostasis. Furthermore, GPR116 ko mouse lines are differently generated. In the mouse line used in our study only the G protein-mediated signaling is fully abolished (Supplementary Fig. 2). Because we still detect significant cell surface expression of the receptor’s N terminus, we cannot exclude *trans*-signaling (N terminus as ligand or interaction partner for other proteins). This is a specific feature of aGPCR which can display *cis*- and *trans*-signaling abilities with different physiological functions^56,57^.

As the reduced amount of insulin content as well as the reduced glucose-induced insulin secretion is contradictory to the results of the glucose tolerance test, we also analyzed plasma insulin and C-peptide content after glucose administration (Fig. 5a, b). Interestingly, we found comparable insulin amounts but significantly reduced C-peptide in GPR116 ko mice. Our further work, therefore, concentrated on the compensating actions regulating plasma insulin. Here, we find reduced IDE activity connected to increased expression of proinsulin in hepatocytes (Fig. 5). Since proinsulin is a known inhibitor of IDE^58^, we conclude that the increased expression of proinsulin in hepatocytes regulates the degradation of insulin resulting in wt-like glucose tolerance.

Furthermore, our immunofluorescence analysis revealed a reduced number of islets and beta cells in GPR116 ko mice which likely accounts for the reduced insulin content and, subsequently, insulin secretion. This phenotype is clearly independent of GPR116 function in delta cells. The reduction in insulin might be the cause for the observed increased fasting blood glucose levels in whole-body ko mice, however, with *Gpr116* expressed in several tissues including liver, we cannot exclude a non-islet cause for the increased fasting blood glucose levels. However, in situ hybridization revealed *Gpr116* mRNA expressed in *Cd34* positive cells. High expression of *Gpr116* has been shown for endothelial cells in general^27^ but also for islet endothelial cells^59^. Furthermore, *Gpr116* is amongst the 10 highest expressed GPCRs during islet development (E18)^14^ indicating that it is involved in developmental processes. Interestingly, when analyzing insulin expression in pancreas of newborns at postnatal day 1, we observed increased insulin expression in ko animals (Fig. 6e, j). Such high insulin levels during islet development have been shown to induce apoptosis in beta cells^60^, which may explain the reduced number of insulin-positive cells in GPR116-deficient animals. Therefore, one can assume that GPR116 is important during islet development mediating an anti-apoptotic effect on beta cells. Taking all data together, we clearly demonstrate that delta-cell expressed GPR116 activation induces secretion of somatostatin. Furthermore, we show that whole-body lack of GPR116 leads to a mild phenotype of increased blood glucose levels caused by increased gluconeogenesis. However, with expression of GPR116 in several tissues, we currently cannot clarify which tissue-specific function of GPR116 leads to this phenotype. Similarly, we find an effect of GPR116 onto islet development. However, at the current stage we cannot distinguish how GPR116 is involved in this process. We could speculate that loss of the receptor in an endocrine precursor cell or the lack in endothelial cells is responsible for the observed phenotype, however, it does not necessarily need to be located in one of these cell types.

In summary, our data implies that GPR116 has different functions in pancreatic islets: in adult pancreatic islets, delta cell-expressed GPR116 can augment somatostatin secretion after receptor activation. In addition, GPR116 is required for the development of fully functional islets by influencing islet number, amount of beta cells as well as insulin expression, and subsequently, insulin content. This strengthens the idea that GPR116 has a dual function in fine-tuning pancreatic islet development and endocrine functionality.

## Methods

### Material

All standard chemicals were purchased from Sigma-Aldrich Chemie GmbH or C. Roth GmbH + Co. KG. If not mentioned otherwise, reagents, cell culture materials, and kits were obtained from Thermo Fisher Scientific. Primers were synthesized from Seqlab or Thermo Fisher Scientific. Peptide synthesis was carried out by the Core Unit Peptide Technology^22^.

### Ca^2+^ imaging

Fura-2-based Ca^2+^ imaging was performed in transfected HEK293T cells (Supplementary Experimental Procedures) and intact pancreatic islets using a monochromator-based imaging system, and the imaging software TILLvisION 4.0 (T.I.L.L. Photonics). Emitted fluorescence (excited at 340 and 380 nm) was acquired at intervals of 2 seconds and corrected for background fluorescence. HEK293T cells and islets were loaded with 5 µM fura-2 AM in standard bath solution containing 140 mM NaCl, 10 mM HEPES, 5 mM KCl, 2 mM CaCl_2_, 1 mM MgCl_2_, and 3 mM glucose for 30 or 60 min. HEK293T cells (25,000 cells/well) were seeded into 24-well plates on poly-L-lysine coated glass cover slips and were transiently transfected with plasmids encoding for GPR116 and GFP the next day. Ca^2+^ imaging was carried out 72 h post transfection. HEK293T cells were exposed to p116sc, p116 (1 mM each), and ATP (100 µM) in standard bath solution. Pancreatic mouse islets (3 - 4 islets per coverslip) were transferred one day after preparation into 12-well plates on poly-L-lysine coated glass cover slips. 48 h after seeding islets were exposed to 1 mM p116sc, 1 mM p116, and 100 µM carbachol (CCh) in standard bath solution.

### RNA isolation, reverse transcriptase PCR, and quantitative real-time PCR

RNA was isolated from pelleted cells or 80–180 pancreatic islets with ReliaPrep^TM^ RNA Cell Miniprep System (Promega) or PicoPure^TM^ RNA Isolation Kit, respectively. Tissue RNA from pancreas and liver was isolated using SV Total RNA Isolation System (Promega) according to the manufacture’s protocol. RNA concentration and quality were determined using the NanoDrop technology (Peqlab). Reverse transcription was carried out with Superscript II Reverse Transcriptase using oligo (dT) and random hexamer primers following the manufacturer’s instructions.

Quantitative real-time PCR (qPCR) was performed by Platinum SYBR Green qPCR SuperMix-UDG with the MxPro3000 instrument (Agilent). The qPCR setup was as follows: each reaction (20 µl) contained of 10–30 ng cDNA, 10 µM primers, 6.25 µl SYBR Green and 0.5 µl of reference dye ROX (1:10 diluted in water). All samples were run in triplicates using the following protocol: 2 min at 50 °C, 2 min at 95 °C, 45 cycles of 95 °C for 15 s and 60 °C for 30 s. Subsequently, melt curves were recorded (50–95 °C, 0.5 °C increment, 10 s/step). Quantitative PCR primer pairs are summarized in Supplementary Table 1. Measured Ct values were normalized to the reference gene (β-actin, *Actb*) to calculate ΔCt values as relative expression for the genes of interest.

### Animals

Mice were bred under specific pathogen-free conditions on 12 h light/12 h dark cycle, 21 °C, and 55% humidity with ad libitum access to food and water. All experiments were conducted in accordance with European Directive 2010/63/EU on the protection of animals used for scientific purposes and were performed with permission from the Animal Care and Use Committee (ACUC# T24/16; T19/18; TVV02/15; TVV43/18) and the Government of the State of Saxony, Germany. We have complied with all relevant ethical regulations for animal use. All mice were between 10 and 16 weeks and were matched to age and sex.

### Mice strains and genotyping

MIP-GFP (B6.Cg-Tg(Ins1-EGFP)1Hara/J) express GFP under the control of mouse insulin 1 promoter leading to GFP expression in pancreatic beta cells^61^. Sst-Cre (B6N.Cg-Ssttm2.1(cre)Zjh/J) are knock-in animals with Cre recombinase expression in somatostatin-positive cells. Both mice strains were obtained from Jackson laboratories (#006864 and #018973).

The generation of GPR116 ko and GPR116-floxed mice has been described previously^23^. Delta cell-specific GPR116 deletion was obtained by crossing GPR116-floxed and Sst-Cre mice. Thereby, mice heterozygous for Cre recombinase and homozygous for GPR116-floxed represent ko animals. The corresponding wt mice also contain heterozygous Cre recombinase but the wt background at the GPR116 locus.

Tail biopsies were digested overnight with proteinase k (500 µg/ml) in lysis buffer (1 M Tris-HCl, 10% SDS, 0.5 mM EDTA, 5 M NaCl, pH 8). An ethanol precipitation protocol was used for extraction of genomic DNA followed by genotyping PCR.

MIP-GFP mice were genotyped using 5’-AAGTTCATCTGCACCACCG and 5’-TCCTTGAAGAAGATGGTGCG by applying the following protocol: denaturation at 94 °C for 3 min, followed by 30 cycles of 94 °C for 30 s, 60 °C for 45 s, and 72 °C for 60 s and a final amplification step of 72 °C for 10 min. Genotyping of GPR116 mice was performed by PCR with following primers: 5’-GTGACAAACCCTGTAGACA, 5’-AAACCCAAACAAGAGGAGGA, and 5’-TACTTGTGTGATGGGGACAT. PCR protocol was used for genotyping as follows: initial denaturation at 94 °C for 5 min, followed by 30 cycles of 94 °C for 30 s, 62 °C for 45 s, and 72 °C for 30 s and a final amplification step of 72 °C for 10 min. For genotyping of GPR116-floxed mice genomic DNA was amplified using 5’-GTGACAAACCCTGTAGACA and 5’-AGAAAATCTCTCCCCTGC for 35 cycles (95 °C for 4 min initial denaturation; 95 °C for 30 s, 52 °C for 45 s, and 72 °C for 90 s; 72 °C for 10 min for final amplification). Determination of Sst-Cre was performed with the following primers: 5’-GGGCCAGGAGTTAAGGAAGA, 5’-TCTGAAAGACTTGCGTTTGG, and 5’-TGGTTTGTCCAAACTCATCAA using a touch-down protocol (95 °C for 5 min initial denaturation; 94 °C for 45 s, 10 cycles 65 °C to 60 °C for 45 s followed by 28 cycles of 60 °C, and 72 °C for 90 s; 72 °C for 10 min).

### Islet isolation

Pancreatic islets were isolated from mice sacrificed by cervical dislocation. Collagenase p solution (0.5 mg/ml) was dissolved in ice-cold Krebs-Ringer buffer (KRB: 115 mM NaCl, 4.7 mM KCl, 1.2 mM KH_2_PO_4_, 1.2 mM MgSO_4_, 2.56 mM CaCl_2_, 20 mM NaHCO_3_, 10 mM Hepes, 0.1% BSA, 5 mM glucose, pH 7.3) and was injected via the common bile duct into pancreas. Afterwards, distended pancreas was digested in shaking water bath at 37 °C for 12–15 min and isolated islets were cultured in RPMI + 10% FBS and 100 units/ml penicillin, and 100 μg/ml streptomycin overnight at 37 °C and 5% CO_2_ in a humidified atmosphere. For Ca^2+^ imaging, islets were cultured up to 3 days.

### Islet stimulation and hormone determination

Equilibration of five islets was performed in KRB containing 2.8 mM glucose for 30 min at 37 °C in 96-well plates. If not mentioned otherwise, islets were stimulated with peptide solution or respective controls in KRB for 30 min at 37 °C. Islets were lysed with acid ethanol and supernatant as well as lysates kept frozen at −20 °C until measurement. To measure insulin the AlphaLISA technology (Perkin Elmer) was used according to the manufacturer’s protocol in 384-well OptiPlate microplates (Perkin Elmer) with the EnVision Multilabel Reader (Perkin Elmer). Somatostatin amounts were determined using either AlphaLISA (Perkin Elmer) or ELISA (Phoenix Pharmaceuticals). For reliable measurement protocol adaptions were necessary. For AlphaLISA these were as follows: increasing sample volumes to 35 µl/well, reduced bead volume of 5 µl/well, and an extended incubation period with donor beads of 90 min. For the ELISA measurement the sample incubation time was extended to over-night and the biotinylated peptide was diluted 1:5 to achieve reliable results.

### Combined fluorescence in situ hybridization and immunolabeling

The RNAscope Multiplex Fluorescent Reagent Kit v2 (Advanced Cell Diagnostics, ACD) was used to colocalize multiple RNAs and antigens on the same section. Pancreas samples were immediately fixed in 4% formalin for 24 hours and embedded in paraffin. 10-µm-thick sections were prepared from the paraffin blocks and mounted on Superfrost® slides. For dual labeling of specific *Gpr116* and somatostatin RNA, a mixture of 1:50 diluted commercially purchased probes for *Gpr116* (318021-C2, ACD) and somatostatin (404631-C3, ACD) was prepared. The freshly diluted probes were then incubated for 2 hours at 40 °C in a manual HybEZ™ II assay hybridization system (ACD) on the organ sections. This was followed by two consecutive amplification steps and labeling of the probes with single Opal fluorophores at a dilution of 1:750 (Akoya Biosciences). *Gpr116* was labeled with Opal 570 (OP-001003) and somatostatin was labeled with Opal 690 (OP-001006). Between each RNA labeling step, the sections were thoroughly washed with wash buffer. After completion of RNA detection, either insulin or glucagon was co-detected by immunolabeling. Prior to antigen labeling, sections were treated with blocking buffer (0.3% Tris / 0.3% Triton X-100 / 5% NGS in PBS). Immunostaining with diluted primary rabbit polyclonal antibodies against insulin (3014 S, Cell Signaling Technologies, 1:1000) and glucagon (2760 S, Cell Signaling Technologies, 1:400) was performed overnight at 4 °C. Binding of the primary antibodies was labeled after washing in PBS with a 1:250 diluted secondary Alexa-488-conjugated goat anti-rabbit antibody (A11034, Invitrogen) at room temperature for 1 hour. After another wash, DAPI nuclear counterstaining (ACD) was performed prior to embedding the sections in Dako fluorescent mounting medium (Aligent). LSM 700 confocal laser scanning microscopy was used to evaluate and document the results of fluorescent in situ hybridization and immunolabeling.

### Immunofluorescence assessment of islet tissue

Semiautomated quantification of pancreatic endocrine cell masses was essentially performed as described by Golson and coworker^62^ and involved standardized systematic tissue preparation, high-resolution slide scanning and computerized image analysis. In brief, pancreata were removed in total from the retroperitoneal space from lateral to medial under binocular microscopic view. The dissected pancreata were weighted, spread out longitudinally in cassettes, fixed in 4% formaldehyde overnight, dehydrated, and embedded in paraffin. The paraffin blocks were cut 5 µm thick in 10 series each containing sections a 250 µm distance to each other. By that series with 10 to 12 sections per pancreas were yielded. The sections in a series were dewaxed and either HE-stained or indirect immunofluorescence double*-*labeled for the detection of somatostatin in combination with insulin. Prior to antibody incubation sections were microwaved for antigen retrieval in 10 mM citrate buffer (pH 6.0) for 5 min, and exposed to 3% normal goat serum (NGS) containing 0.3% Triton X-100 for 1 hour for reduction of background staining. Primary antibody incubation was performed with a cocktail including 1:400 diluted rat anti-somatostatin (Abcam) and 1:2,000 diluted rabbit anti insulin (Cell Signaling Technologies) or 1:400 diluted rabbit anti glucagon (Cell Signaling Technologies) antibodies. The primary antibody cocktail was applied in PBS supplemented with 0.5% NGS and 0.03% Triton-X 100 in a moist chamber at 4 °C overnight. Primary antibody binding was probed with a secondary antibody cocktail containing Alexa 568 goat anti rat and Alexa 488 goat anti rabbit antibodies both at a 1:250 dilution in a moist atmosphere at 25 °C for 30 min. After immunofluorescence labeling, sections were thoroughly washed, nuclei stained with 4′,6-diamidino-2-phenylindole (DAPI), coverslipped in Roti®*-*Mount FluorCare mounting medium and hardened overnight.

Double immunofluorescence-stained sections were fully digitized at 20x magnification using a digital slide scanner (Pannoramic Scan II, 3D HISTECH Ltd.) equipped with a quad band (DAPI/FITC/TRITC/Cy5) filter set. Images of the sections were manually exported from slide scanner data sets (Pannoramic Viewer, Version 1.15.4, 3D HISTECH Ldt.) as TIFF images with pixel dimensions of 0.65 µm. Perimeters of pancreatic islets were manually drawn onto the exported images (GIMP 2.10.2) and exported as a separate image containing all islet masks of the respective fluorescence image. Image analysis was performed with Mathematica (Version 11.1, Wolfram Research, Inc.). Fluorescence images were imported and split into separate color channels. Color correction of red and green image channels was performed by subtracting one from the other. This procedure resulted in red and green images containing only relevant signals without significant background fluorescence. Matching islet mask images were imported and all present islet regions were identified and counted. Image sections of each islet region were extracted from the color corrected images and separately submitted to image segmentation. Green channels (insulin) were segmented using Otsu’s (cluster variance maximization) thresholding method^63^, while red channels (somatostatin) were segmented using Kapur’s (histogram entropy minimization) thresholding method^64^. Finally, the area of each islet region and its segmented red and green cell content was computed.

### Functional in vivo studies

Glucose and pyruvate tolerance tests were carried out with mice fasted overnight (15 - 18 h). Blood glucose levels were measured before intraperitoneal injection of glucose (10 µl/g body weight of 20% glucose solution, Glucosteril, Fresenius Kabi GmbH) or pyruvate (10 µl/g body weight of 10% pyruvate solution). Blood glucose levels were determined at the indicated time points after injection. For insulin tolerance tests mice were fasted for 6 h and blood glucose levels determined. Insulin (1 mU/g body weight human Insuman Rapid, Sanofi) was injected intraperitoneally and blood glucose levels determined at the indicated time points.

### Hepatocyte isolation and determination of IDE activity

Primary hepatocytes were isolated from male and female GPR116 ko mice and wt siblings. Isolation of liver cells is based on an in vivo perfusion method described by Seglen in the 1970s^65^ and used today with modifications^66–68^. Both the portal vein and *vena cava* are cannulated to perfuse the liver with buffer for calcium depletion and blood washing, followed by perfusion with collagenase. This two-step procedure allows the matrix of the liver parenchyma to be dissolved. The closed liver capsule allows the entire liver to be removed from the animal after perfusion and transferred to buffer. After rupture of the capsule, the liver cells, both hepatocytes and NPCs, are in suspension. To ensure the highest possible purity of the hepatocytes, the NPC fraction should be separated as completely as possible by differential centrifugation steps. IDE activity was determined using SensoLyte® 520 IDE Activity Assay (AnaSpec Inc.) according to the manufactures’ protocol. In brief, 1 × 10^6^ hepatocytes were washed with phosphate-buffered saline and lysed in 1 ml assay buffer. After 15 min incubation on ice, cells were centrifuged and the supernatant stored at -70 °C until use. Then, the supernatant was mixed with IDE substrate solution, incubated for 30 min at 37 °C, and fluorescence intensity was measured at the EnVision Multilabel Reader (Ex/Em = 490 nm/520 nm).

### Statistics and reproducibility

For statistical and graphical analyses Prism version 9 (GraphPad Software Inc., San Diego, CA) was used. Comparing two groups, statistical significance was analyzed using the two-tailed *t* test, comparing two groups at multiple time points an unpaired multiple *t* test corrected for multiple testing using the Holm-Šídák method was applied. When comparing several datapoints to one reference one-way ANOVA with Dunnet’s multiple comparison test was performed. The applied tests are given in the figure legends. *P ≤ 0.05; **P ≤ 0.01; ***P ≤ 0.001.

### Reporting summary

Further information on research design is available in the Nature Portfolio Reporting Summary linked to this article.

### Supplementary information

Supplemental Material

Reporting Summary

## Data Availability

Data supporting the findings of this study have been deposited at Zenodo (10.5281/zenodo.10459692)^69^ and are publicly available as of the date of publication. All other information is available from the corresponding author on reasonable request.
