# Supplementary material for: The adhesion GPCR GPR116/ADGRF5 has a dual function in pancreatic islets regulating somatostatin release and islet development
Source: Commun Biol. 2024 Jan 16;7:104. doi: 10.1038/s42003-024-05783-9 (PMC10791652; doi:10.1038/s42003-024-05783-9)
Supplement: Supplementary file 1 — Supplemental Material [file 42003_2024_5783_MOESM1_ESM.pdf]

Supplementary Information

Title

The adhesion GPCR GPR116/ADGRF5 has a dual function in pancreatic islets regulating somatostatin release and islet development

Authors

Juliane Röthe[1], Robert Kraft[2], Albert Ricken[3], Isabell Kaczmarek[1], Madlen Matz-Soja[4,5], Karsten Winter[3], André Nguyen Dietzsch[1], Julia Buchold[1], Marie-Gabrielle Ludwig[6], Ines Liebscher[1], Torsten Schöneberg[1], and Doreen Thor[1]*

Affiliations

[1]Rudolf Schönheimer Institute of Biochemistry, Medical Faculty, Leipzig University, Leipzig, Germany

[2]Carl-Ludwig-Institute for Physiology, Medical Faculty, Leipzig University, Leipzig, Germany

[3]Institute of Anatomy, Medical Faculty, Leipzig University, Leipzig, Germany

[4]Medical Department II – Gastroenterology, Hepatology, Infectious Diseases, Pneumology, University Medical Center, Leipzig, Germany

[5]Division of Hepatology, Clinic and Polyclinic for Oncology, Gastroenterology, Hepatology, Infectious Diseases, and Pneumology, University Hospital, Leipzig, Germany

[6]Novartis Institutes for Biomedical Research, Basel, Switzerland

Contact information

*To whom correspondence should be addressed:

Doreen Thor, Telephone: +49 341 9722177

E-mail: doreen.thor@medizin.uni-leipzig.de

Supplementary Results

Stachel peptide-mediated GPR116 activation

Prior to the activation of endogenously expressed GPR116, we tested the specificity of p116 and p116sc in transfected cells (Supplementary Figs. 1c – g). GPR116 is activated by p116 resulting in a significant increase in the intracellular IP_1 concentration via activation of the $G_{q/11}$ protein/phospholipase C-signaling pathway, whereas the control peptide (p116sc) did not induce a response (Supplementary Fig. 1c). In GPR116-transfected cells, the stimulation with p116 resulted in a 12-fold activation of NFAT reporter gene compared to mock-transfected cells whereas p116sc did not show any effect upon receptor activation (Supplementary Fig. 1d). In line with a phospholipase C-dependent mechanism, intracellular Ca^{2+} transients were induced by p116 or by activation of endogenous purinergic receptors with ATP in GPR116-transfected HEK293T cells, while p116sc failed to evoke Ca^{2+} signals. Mock-transfected cells did neither respond to p116 nor p116sc, whereas ATP strongly increased intracellular Ca^{2+} levels (Supplementary Figs. 1e, f, and g). These data confirm that GPR116 signals via $G\alpha_{q/11}$ protein/phospholipase C/Ca^{2+} pathway upon stimulation with p116[1–3].

Characterization of GPR116 ko construct

The constitutive GPR116 ko mouse strain was derived from exon-17-floxed mouse strain (*Gpr116*$^{f/f}$)[4]. Exon 17 of *Gpr116* encodes for most of the 7-transmembrane helix domain mediating G-protein coupling. The same ko mouse line was used to study G-protein signaling of GPR116 in alveolar type II cells[1]. PCR analysis revealed *Gpr116* transcripts with exon 17 deletion (∆exon17-variant). The resulting open-reading frame encodes for a 7-TM-deficient receptor protein still containing the C-terminal half of the transmembrane helix 7 and the C terminus (Supplementary Figs. 2a and 2b). *In vitro* expression of the ∆exon17-variant in COS-7 cells showed a significantly reduced cell surface expression but unchanged total expression of truncated GPR116 compared to the full-length receptor (Supplementary Fig. 2c). This indicates intracellular retention of the receptor mutant but also an obvious portion of a plasma membrane-anchored N terminus of GPR116. However, IP_1 accumulation was completely abolished in cells expressing the mutant GPR116 indicating no functional G-protein coupling *in vitro* (Supplementary Fig. 2d).

Supplementary Experimental Procedures

Cell culture and *in vitro* functional assays

HEK293T and COS-7 cells were cultured in Dulbecco's minimum essential medium (DMEM) supplemented with 10 % fetal bovine serum (FBS), 100 units/ml penicillin, and 100 µg/ml streptomycin at 37 °C and 5 % CO_2 in a humidified atmosphere. Generation of mouse GPR116 plasmid were described previously[2].
Luciferase reporter gene assays and quantification of inositol 1 phosphate (IP_1) were described previously[2]. Briefly, for luciferase reporter gene assays HEK293T cells were co-transfected with receptor plasmid (25 ng/well) and NFAT reporter construct (50 ng/well; PathDetect trans-reporting system) using Lipofectamine^{TM} 2000 according to the manufacturer's protocol. After 24 h HEK293T cells were treated with 2 mM peptide solution or respective controls in phenol red-free DMEM for 5 h at 37 °C. Stimulation was terminated by washing cells once with PBS and addition of 100 µl luciferase assay reagent (SteadyLite, Perkin Elmer). The measurement of fluorescence intensity was performed with the EnVision Multilabel Reader (Perkin Elmer). Measurements of IP_1 were performed in COS-7 with the IP-One Tb kit (Cisbio) according to the manufacturer's protocol. Here, IP_1 accumulation is achieved by LiCl contained in the stimulation buffer which blocks the degradation of IP_1 to myo-inositol. 48 h after transfection COS-7 cells were incubated with IP_1 stimulation buffer containing 1 mM peptide or respective controls. After 30 min cells were lysed. Accumulated IP_1 was determined in cell extracts using ProxiPlate-384 Plus microplates (Perkin Elmer) with the EnVision Multilabel Reader (Perkin Elmer).

Cell surface and total ELISA

All receptor constructs carried an N-terminal HA and a C-terminal FLAG tag to permit for enzyme-linked immunosorbent assay (ELISA) -based receptor detection. Cell surface expression was analyzed using an indirect ELISA. Transiently transfected COS-7 cells were fixed with 4 % formaldehyde and incubated with blocking solution (media + 10 % FBS) for 1 h at 37 °C. ELISA was performed with an anti-HA-peroxidase-conjugated antibody (Roche) as previously described[5].
A sandwich ELISA was used to estimate the total amounts of receptor constructs. After lysis of transfected COS-7 cells the total expression of receptors were analyzed with an anti-FLAG-M2- and an anti-HA-peroxidase-conjugated antibody (Roche) as previously described[6].

Generation of GPR116 ko construct

GPR116 ko construct was amplified from mouse lung cDNA with following primers: 5’-ATCAGCATCCTTGACTTGCT (sense) and 5’-GCAGTAACTAGTCTACTTATCGTCGTCATCCTTGTAATCGTTGAGCAGTGAGTAAG (antisense). The reverse primer contained the FLAG-Tag and an *Spe*I restriction site. The PCR reaction was carried out with the Q5 polymerase (New England Biolabs) following the manufacturer’s instructions. The reaction was initiated with a denaturation at 98 °C for 30 s, followed by 35 cycles of denaturation at 98 °C for 10 s, annealing at 65 °C for 30 s and elongation at 72 °C for 60 s. The final amplification step was performed at 72 °C for 2 min. The PCR product was cut with *Spe*I and *EcoR*I, purified and ligated into the existing GPR116 plasmid (in the mammalian expression vector pcDps).

Re-analysis of publicly available RNA-seq data sets

Gene Expression Omnibus (GEO) database was searched for RNA-seq expression data of sorted pancreatic islets. Two different data sets (GSE760178 and (shown as Data Set A, GSE76017[8] and Data Set B, GSE80673[9]) indicate GSE806739) indicate [8,9] were evaluated regarding the expression of *Gpr116* in alpha-, beta-, and delta cells. The values are given in FPKM (Fragments Per Kilobase Million) or RPKM (Reads Per Kilobase Million) to normalize the expression for sequencing depth and gene length.

111 ## Supplementary figures and tables

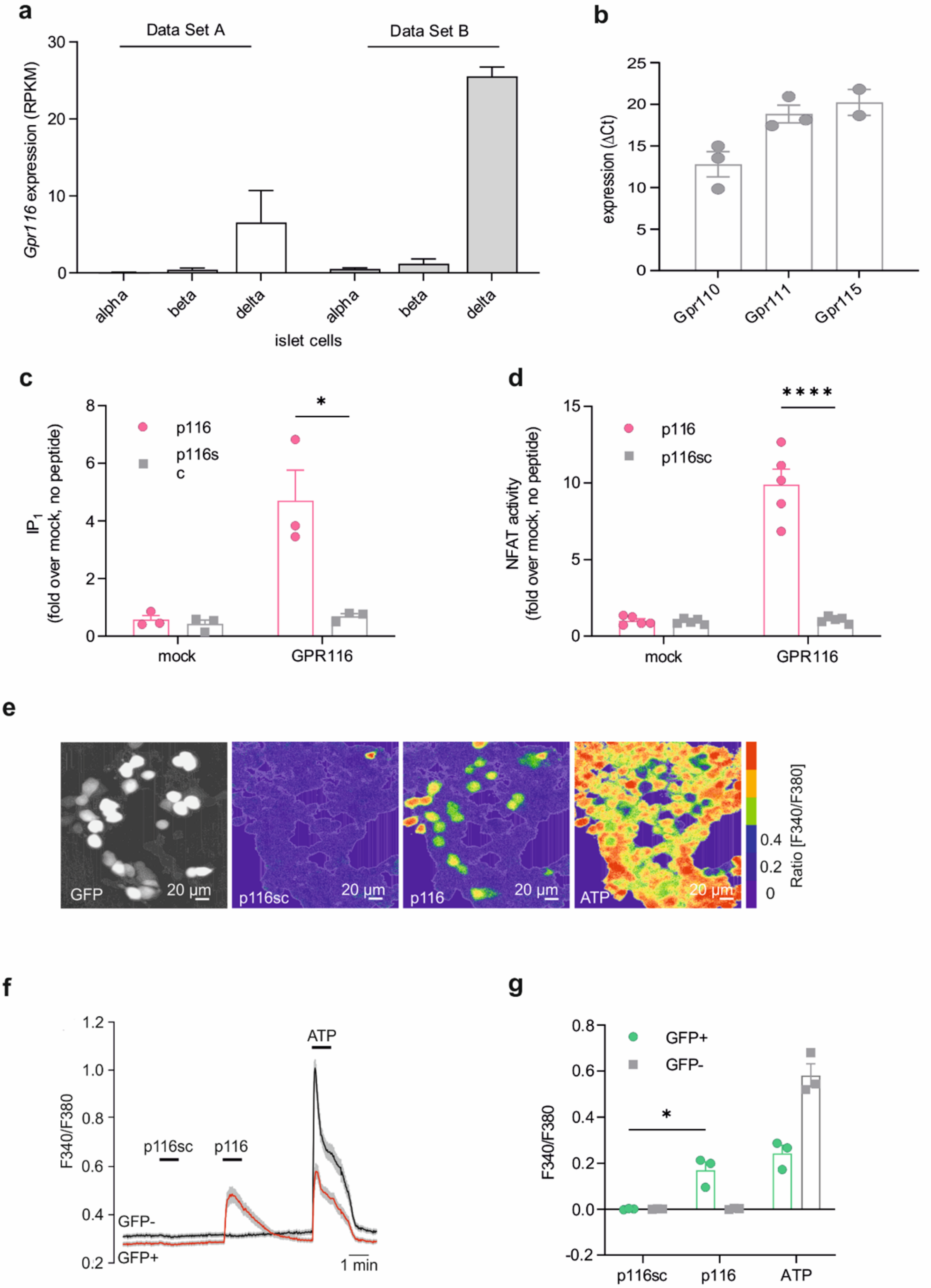

112

Supplementary Figure 1 –*Gpr116* expression in pancreatic islets and *Stachel* peptide-mediated activation.

(**a**) RNA-seq re-analyses of sorted pancreatic islets cells (shown as Data Set A, GSE76017[8] and Data Set B, GSE80673[9]) indicate that GPR116 is predominantly expressed in delta cells. Data are presented as mean of RPKM values ± SD of two to six samples of each cell type.

(**b**) GPR116 belongs to a group of structurally related aGPCR, namely GPR110, GPR111, and GPR115. Since it has been shown that *Stachel*-derived peptides may activate related aGPCRs, we analyzed their expression in pancreatic islets. All three receptors are considerably less expressed compared to GPR116. Quantitative PCR data are presented as mean ± SEM normalized to β-actin of at least two experiments performed in triplicates.

(**c**) IP_1 accumulation was measured after stimulation of GPR116- or mock-transfected COS-7 cells with the GPR116 agonist p116 (1 mM) or the inactive peptide p116sc (1 mM). Data are given as mean ± SEM fold over non-stimulated mock-transfected cells (basal IP_1 level: 242.6 ± 96.1 nM) of three independent experiments performed in triplicates.

(**d**) NFAT-luciferase reporter gene assay indicates a gain of luciferase activity only in GPR116-transfected HEK293T cells after stimulation with 2 mM p116, whereas incubation with 2 mM p116sc does not change luciferase activity. Given is the mean ± SEM as fold over non-stimulated mock-transfected cells (basal: 889.7 ± 409.5 counts) of n = 12 (p116) and n = 5 (p116sc) independent experiments performed in triplicates.

(**e**) Fura 2-based Ca^{2+} imaging experiments were performed in HEK293T cells cotransfected with GPR116 and with GFP as transfection control. Representative images show the fluorescence ratio (F340/F380) after administration of p116sc (1 mM), p116 (1 mM), and ATP (100 µM).

(**f**) Time course of Ca^{2+} in GFP-positive (GFP+) and GFP-negative (GFP-) cells from the same coverslip, as shown in (c). Each trace represents the average Ca^{2+} signal ± SEM from 31 cells.

(**g**) Average Ca^{2+} signals ± SEM are presented as mean delta ratio (F340/F380) ± SEM of three independent experiments each containing 25 to 49 GFP+ and GFP- cells. Only GFP+ cells showed responses to p116 (1 mM). ATP

(100 µM) induced signals in both GFP+ and GFP- cells, whereas p116sc was ineffective.
P values were determined using an unpaired multiple t-test (Holm-Šídák method) (*$P \leq 0.05$, ****$P \leq 0.0001$).

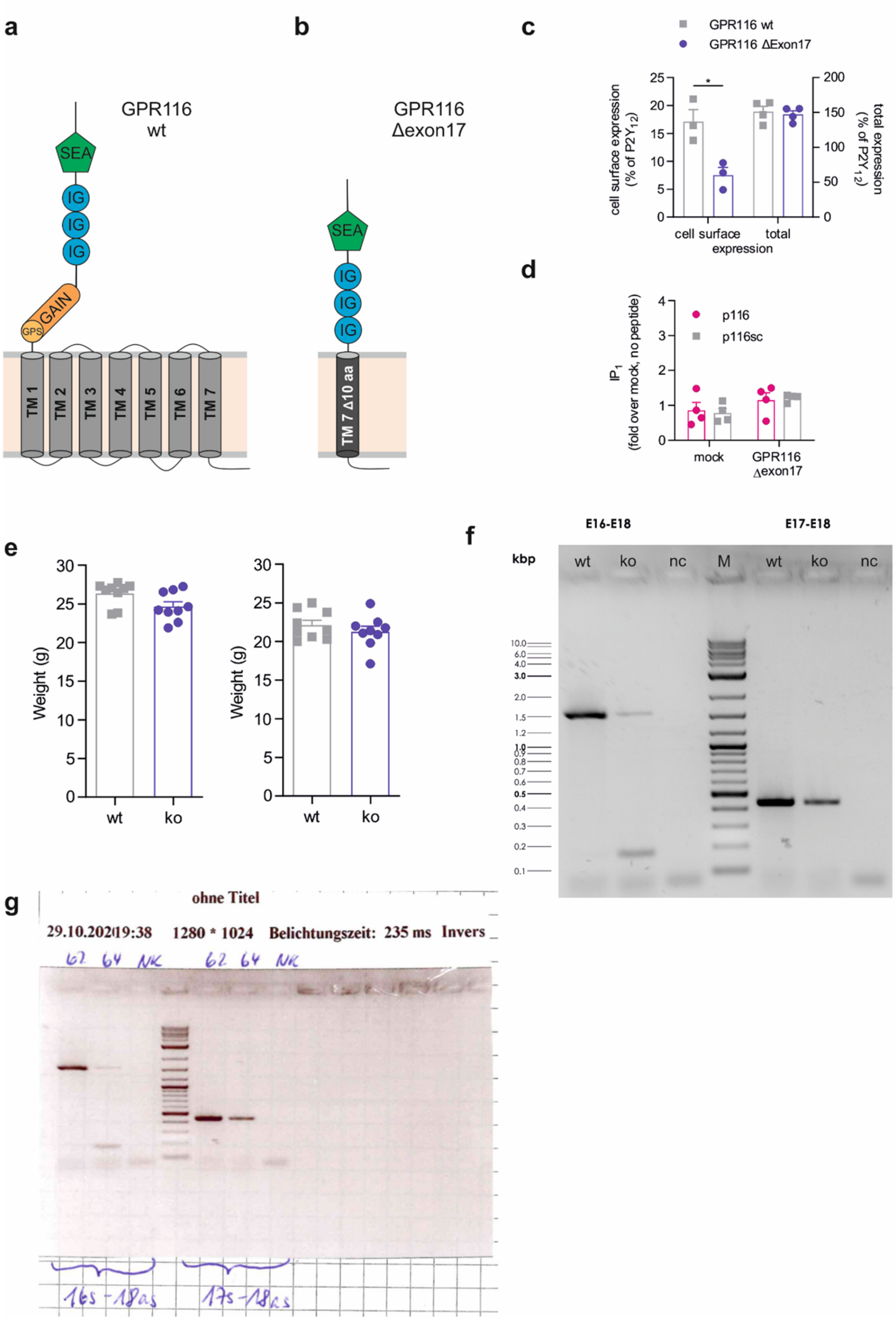
a
GPR116
wt
SEA
IG
IG
IG
GAIN
GPS
TM 1
TM 2
TM 3
TM 4
TM 5
TM 6
TM 7
b
GPR116
Δexon17
SEA
IG
IG
IG
TM 7 Δ10 aa
c
GPR116 wt
GPR116 ΔExon17
cell surface expression (% of P2Y12)
total expression (% of P2Y12)
cell surface
total
expression
d
p116
p116sc
IP1 (fold over mock, no peptide)
mock
GPR116 Δexon17
e
Weight (g)
wt
ko
f
E16-E18
E17-E18
kbp
wt
ko
nc
M
g
ohne Titel
29.10.2020 19:38
1280 * 1024
Belichtungszeit: 235 ms
Invers
62
64
NK
16s - 18as
17s - 18as

Supplementary Figure 2 – Characterization of GPR116$^{\Delta exon17}$ construct.

(**a** & **b**) Schematic depiction of the GPR116 wt receptor and GPR116 ko receptor with deletion of exon 17.

(**c**) Cell surface and total expression of mouse GPR116 wt and GPR116$^{\Delta exon17}$ receptor were detected in transfected COS-7 cells using an indirect or sandwich ELISA system, respectively. While total expression was unchanged, cell surface expression levels of GPR116$^{\Delta exon17}$ were decreased in comparison to the wt receptor, however, cell surface expression was still detectable. Values are given as percentage of $P2Y_{12}$ receptor as positive control (OE readings of $P2Y_{12}$: 1.29 ± 0.13 and 0.913 ± 0.034 for cell surface expression and total expression, respectively) and are shown as mean ± SEM for n = 3 (cell surface expression) and n = 4 (total expression) performed in triplicates. Non-specific OD values of empty vector transfected cells were 0.003 ± 0.001 (cell surface expression) and 0.064 ± 0.004 (total expression).

(**d**) IP_1 concentrations were quantified in mock- and GPR116$^{\Delta exon17}$-transfected COS-7 cells after incubation with p116 (1 mM) and p116sc (1 mM). Data are shown as fold over non-stimulated mock-transfected cells (basal IP_1 level: 207.8 ± 76.1 nM) and given as mean ± SEM of four independent experiments performed in triplicates.

(**e**) No differences in body weight of male (left panel) or female (right panel) between wt and ko mice were observed indicating that metabolic changes are not connected to an increase in body weight. Given is the mean ± SD of ten wt and ko mice.

(**f**) PCR analysis of cDNA from pancreatic islets isolated from delta cell-specific *Gpr116* ko mice reveal partial deletion of *Gpr116*. Two different primer sets were used to amplify cDNA with sense primers located on exon 16 (lane 1 - 3) or exon 17 (lane 5 - 7) and antisense primer located on exon 18. Given that exon 17 is deleted pancreatic delta cells due to Cre expression in somatostatin expressing cells, primer set E16/E18 should yield a smaller fragment, whereas primer set E17/E18 should have reduced amplification due to *Gpr116* expression in other islet cells. For reference, 1 kb Plus DNA Ladder (NEB) was used to identify the size of the PCR products. (**g**) Uncropped image of gel electrophoresis shown in (**f**).

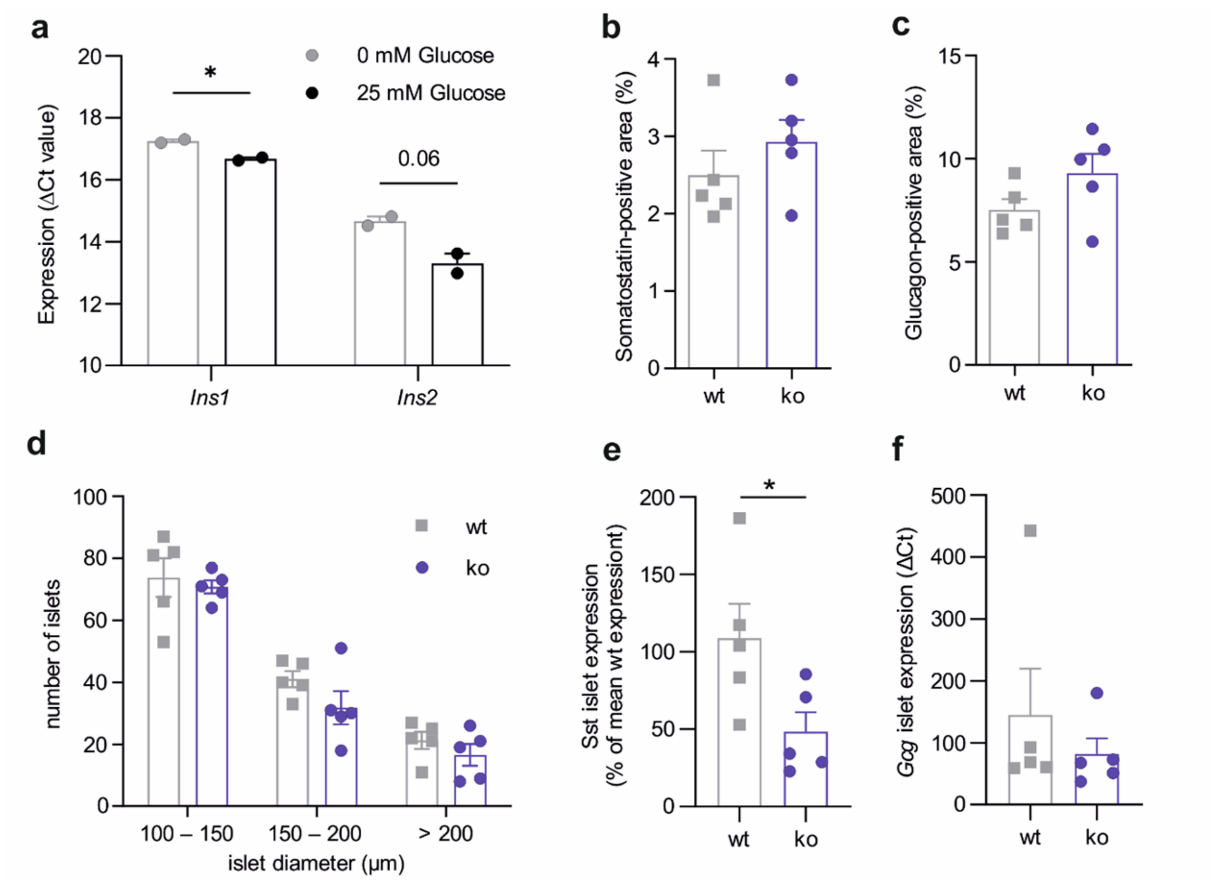

Supplementary Figure 3 – Islet specific changes due to constitutive deficiency of GPR116.

(**a**) Isolated wt hepatocytes were incubated with different glucose concentrations (0 mM *vs*. 25 mM) for 24 hours and insulin expression was determined demonstrating that increased glucose concentration stimulate expression of the insulin genes *Ins1* and *Ins2*.

(**b**) The quantification of somatostatin-positive area is presented as ratio of somatostatin-positive cells to total pancreatic islet area. Given is the mean ± SEM of five wt and ko mice.

(**c**) The quantification of glucagon-positive area is presented as ratio of glucagon-positive cells to total pancreatic islet area. Given is the mean ± SEM of five wt and ko mice.

(**d**) Depending on islet diameter islets were grouped into small (<100 μm) (see Fig. 5f), medium (100 – 150 μm), large (150 – 200 μm), and very large (>200 μm) islets. Islet numbers of each group were counted in all sections of five wt and ko animals and are presented as mean ± SD.

(**e**) Somatostatin expression (*SSt*) was determined in in adult islets from 5 wt and ko animals. Data is given as mean ± SEM normalized to expression of β-actin as reference gene.

(**f**) Glucagon expression (*Gcg*) was determined in in adult islets from 5 wt and ko animals. Data is given as mean ± SEM normalized to expression of β-actin as reference gene.

Statistical significance was tested using a two-tailed unpaired *t* test (*P ≤ 0.05).

Supplementary Table 1: Primer sequences used for qPCR analyses in islets, pancreas, liver, and cell lines.

gene	Primer forward 5'-3'	Primer reverse 5'-3'
mouse *Gpr116*	GAACACGTCTTCTGCCCTCT	TGCCCTTGGTGAACTGTGTC
mouse *Gpr110*	AGAGCAGGAGCTGAGAGCAG	ACGAGCCACAGAAGTCCAAT
mouse *Gpr111*	TGATCTGAAGGGAGACGACA	AGTACAACCAATGGGCAGGA
mouse *Gpr115*	GTGTGAAAAATGAAGCCCTG	CCTGTCTTGGTCTAAACTCA
mouse *Ins1*	GGGGAGCGTGGCTTCTTCTA	CCAAGGTCTGAAGGTCCCCG
mouse *Ins2*	TGACCTTCAGACCTTGGCACTG	GTAGAGGGAGCAGATGCTGGTG
mouse *Ide*	TGGAAAAGGCTACGGGGAAC	ACGTCGATGCCTTCTTGGTT
mouse *Pfkl Iso1*	TGAGGATGGCTGGGAGAACT	TGAACCACCAGATCCTTCACG
mouse *Pfkl Iso2*	CATTGACCGGCATGGAAAGC	TGCAGCCCTGGCTATTCAAA
mouse *Ldha*	GTCCAGCGAAACGTGAACAT	TCCAAGCCACGTAGGTCAAG
mouse *Pklr*	CCGAGATACGCACTGGAGTC	GTGGTAGTCCACCCACACTG
mouse *Pygl*	CCTATGGCTACGGCATTCGT	TCTCCCAAGGGTTTCCATGC
mouse *Fbp1*	AGTCGTCCTACGCTACCTGT	TGGTTCCGATGGACACAAGG
mouse *Gys2*	TCACCGTTTTCTCTGAACCACT	CTGTTGGTCTGCATCAGGGT
mouse *Actb*	GCTCTTTTCCAGCCTTCCTT	CGGATGTCAACGTCACACTT
human GPR116	GAACACTTCCTCCGCCCTCT	TTCCAGACTTGAACCCTGTC
human ACTB	GCACTCTTCCAGCCTTCCTT	CGGATGTCCACGTCACACTT
rat *Gpr116*	GAACACATCCTCTGCCCTCT	TGCCCTTGGTGAACTGTGTC
rat *Actb*	GCTCTCTTCCAGCCTTCCTT	CGGATGTCAACGTCACACTT

Supplementary References

1. Brown, K. *et al.* Epithelial Gpr116 regulates pulmonary alveolar homeostasis via Gq/11 signaling. *JCI insight* **2,** e93700; 10.1172/jci.insight.93700 (2017).
2. Demberg, L. M. *et al.* Activation of adhesion G protein-coupled receptors. Agonist specificity of *Stachel* sequence-derived peptides. *J. Biol. Chem.* **292,** 4383–4394; 10.1074/jbc.M116.763656 (2017).
3. Tang, X. *et al.* GPR116, an adhesion G-protein-coupled receptor, promotes breast cancer metastasis via the Gαq-p63RhoGEF-Rho GTPase pathway. *Cancer Res.* **73,** 6206–6218; 10.1158/0008-5472.CAN-13-1049 (2013).
4. Bridges, J. P. *et al.* Orphan G protein-coupled receptor GPR116 regulates pulmonary surfactant pool size. *Am. J. Respir. Cell Mol. Biol.* **49,** 348–357; 10.1165/rcmb.2012-0439OC (2013).
5. Stäubert, C., Broom, O. J. & Nordström, A. Hydroxycarboxylic acid receptors are essential for breast cancer cells to control their lipid/fatty acid metabolism. *Oncotarget* **6,** 19706–19720; 10.18632/oncotarget.3565 (2015).
6. Römpler, H., Yu, H.-T., Arnold, A., Orth, A. & Schöneberg, T. Functional consequences of naturally occurring DRY motif variants in the mammalian chemoattractant receptor GPR33. *Genomics* **87,** 724–732; 10.1016/j.ygeno.2006.02.009 (2006).
7. Hara, M. *et al.* Transgenic mice with green fluorescent protein-labeled pancreatic beta -cells. *American journal of physiology. Endocrinology and metabolism* **284,** E177-83; 10.1152/ajpendo.00321.2002 (2003).
8. Adriaenssens, A. E. *et al.* Transcriptomic profiling of pancreatic alpha, beta and delta cell populations identifies delta cells as a principal target for ghrelin in mouse islets. *Diabetologia* **59,** 2156–2165; 10.1007/s00125-016-4033-1 (2016).
9. DiGruccio, M. R. *et al.* Comprehensive alpha, beta and delta cell transcriptomes reveal that ghrelin selectively activates delta cells and promotes somatostatin release from pancreatic islets. *Molecular metabolism* **5,** 449–458; 10.1016/j.molmet.2016.04.007 (2016).
