# Supplementary material for: The adhesion GPCR GPR116/ADGRF5 has a dual function in pancreatic islets regulating somatostatin release and islet development
Source: Commun Biol. 2024 Jan 16;7:104. doi: 10.1038/s42003-024-05783-9 (PMC10791652; doi:10.1038/s42003-024-05783-9)
Supplement: Supplementary file 2 — Reporting Summary [file 42003_2024_5783_MOESM2_ESM.pdf]

Corresponding author(s): Doreen Thor

Last updated by author(s): Dec 20, 2023

Reporting Summary

Nature Portfolio wishes to improve the reproducibility of the work that we publish. This form provides structure for consistency and transparency in reporting. For further information on Nature Portfolio policies, see our Editorial Policies and the Editorial Policy Checklist.

Statistics

For all statistical analyses, confirm that the following items are present in the figure legend, table legend, main text, or Methods section.

n/a	Confirmed	
☐	☒	The exact sample size (*n*) for each experimental group/condition, given as a discrete number and unit of measurement
☐	☒	A statement on whether measurements were taken from distinct samples or whether the same sample was measured repeatedly
☐	☒	The statistical test(s) used AND whether they are one- or two-sided<br>*Only common tests should be described solely by name; describe more complex techniques in the Methods section.*
☐	☒	A description of all covariates tested
☐	☒	A description of any assumptions or corrections, such as tests of normality and adjustment for multiple comparisons
☐	☒	A full description of the statistical parameters including central tendency (e.g. means) or other basic estimates (e.g. regression coefficient) AND variation (e.g. standard deviation) or associated estimates of uncertainty (e.g. confidence intervals)
☐	☒	For null hypothesis testing, the test statistic (e.g. *F*, *t*, *r*) with confidence intervals, effect sizes, degrees of freedom and *P* value noted<br>*Give P values as exact values whenever suitable.*
☒	☐	For Bayesian analysis, information on the choice of priors and Markov chain Monte Carlo settings
☒	☐	For hierarchical and complex designs, identification of the appropriate level for tests and full reporting of outcomes
☒	☐	Estimates of effect sizes (e.g. Cohen's *d*, Pearson's *r*), indicating how they were calculated

Our web collection on statistics for biologists contains articles on many of the points above.

Software and code

Policy information about availability of computer code

Data collection	Bio-Rad CFX Maestro 2.3
Data analysis	GraphPad Software Prism version 9 and version 10

For manuscripts utilizing custom algorithms or software that are central to the research but not yet described in published literature, software must be made available to editors and reviewers. We strongly encourage code deposition in a community repository (e.g. GitHub). See the Nature Portfolio guidelines for submitting code & software for further information.

Data

Policy information about availability of data

All manuscripts must include a data availability statement. This statement should provide the following information, where applicable:

- Accession codes, unique identifiers, or web links for publicly available datasets
- A description of any restrictions on data availability
- For clinical datasets or third party data, please ensure that the statement adheres to our policy

Data supporting the findings of this study have been deposited at Zenodo (10.5281/zenodo.10397673) and are publicly available as of the date of publication.

Research involving human participants, their data, or biological material

Policy information about studies with human participants or human data. See also policy information about sex, gender (identity/presentation), and sexual orientation and race, ethnicity and racism.

Reporting on sex and gender	We do not report data on human participants.
Reporting on race, ethnicity, or other socially relevant groupings	We do not report data on human participants.
Population characteristics	We do not report data on human participants.
Recruitment	We do not report data on human participants.
Ethics oversight	We do not report data on human participants.

Note that full information on the approval of the study protocol must also be provided in the manuscript.

Field-specific reporting

Please select the one below that is the best fit for your research. If you are not sure, read the appropriate sections before making your selection.

☒ Life sciences ☐ Behavioural & social sciences ☐ Ecological, evolutionary & environmental sciences

For a reference copy of the document with all sections, see nature.com/documents/nr-reporting-summary-flat.pdf

Life sciences study design

All studies must disclose on these points even when the disclosure is negative.

Sample size	Sample size calculation was performed upon application for permission from the Animal Care and Use Committee as this is requested for the permission. Thereto, G power 3 was used and sample size calculated comparing independent means (two groups).
Data exclusions	We did not exclude data.
Replication	We give the number of independent performed experiments in the figure legends. All experiments were replicated.
Randomization	Randomization was not performed. Animals were assigned to the group according to their genotype. In cell culture experiments, cells / islets were distributed from a cell suspension to the individual wells.
Blinding	Blinding was not performed. In cell culture experiments, cells were seeded from a cell suspension and treated with the indicated substances.

Reporting for specific materials, systems and methods

We require information from authors about some types of materials, experimental systems and methods used in many studies. Here, indicate whether each material, system or method listed is relevant to your study. If you are not sure if a list item applies to your research, read the appropriate section before selecting a response.

Materials & experimental systems

n/a	Involved in the study	
☐	☒	Antibodies
☐	☒	Eukaryotic cell lines
☒	☐	Palaeontology and archaeology
☐	☒	Animals and other organisms
☒	☐	Clinical data
☒	☐	Dual use research of concern
☒	☐	Plants

Methods

n/a	Involved in the study	
☒	☐	ChIP-seq
☒	☐	Flow cytometry
☒	☐	MRI-based neuroimaging

Antibodies

Antibodies used	anti-insulin-AB (3014S, CST); anti-glucagon-AB (2760S, CST); Alexa-488-con. goat anti-rabbit-AB (A11034, Invitrogen); anti-somatostatin-AB (ab30788, Abcam); Alexa-568-con. goat anti-rat-AB (A11077, Invitrogen); anti-HA-POD-AB (12013819001, Roche)

Validation	Antibodies were validated by the vendor. For cell culture, mock-transfected cells were used as negative control.

Eukaryotic cell lines

Policy information about cell lines and Sex and Gender in Research

Cell line source(s)	HEK293T (ATCC); COS-7 (ATCC); aTC-1 (ATCC); MIN6 (Dr. J. Wess, NIH, NIDDK); INS1 (Sigma-Aldrich), QGP-1 (Dr. K. Hill, Leipzig University), pancreatic mouse islets (primary isolated, male and female)
Authentication	Cell lines were not authenticated.
Mycoplasma contamination	Cell lines were negative for mycoplasma and were tested on a monthly basis using MycoAlert® PLUS (Lonza, LT07-710)
Commonly misidentified lines (See ICLAC register)	none

Animals and other research organisms

Policy information about studies involving animals; ARRIVE guidelines recommended for reporting animal research, and Sex and Gender in Research

Laboratory animals	Mouse strains: MIP-GFP (B6.Cg-Tg(Ins1-EGFP)1Hara/J); Sst116 (C-GPR116TM2Npa-floxed x B6N.Cg-Ssttm2.1(cre)Zjh/J); Gpr116ko (C-GPR116TM2Npa); Age: 14 to 16 weeks
Wild animals	None were used.
Reporting on sex	Metabolic characterization was performed for male mice. Body weight was measured for both sexes. Islet isolation was performed for both sexes.
Field-collected samples	None were used.
Ethics oversight	The necessary permissions for animal experiments were issued by Animal Care and Use Committee (ACUC# T24/16; T19/18; TVV02/15; TVV43/18) and the Government of the State of Saxony (Germany).

Note that full information on the approval of the study protocol must also be provided in the manuscript.

Plants

Seed stocks	none were used
Novel plant genotypes	none were used
Authentication	none were used
